# Nsp9 and Nsp10 Contribute to the Fatal Virulence of Highly Pathogenic Porcine Reproductive and Respiratory Syndrome Virus Emerging in China

**DOI:** 10.1371/journal.ppat.1004216

**Published:** 2014-07-03

**Authors:** Yan Li, Lei Zhou, Jialong Zhang, Xinna Ge, Rong Zhou, Huaguo Zheng, Gang Geng, Xin Guo, Hanchun Yang

**Affiliations:** Key Laboratory of Animal Epidemiology and Zoonosis of the Ministry of Agriculture, College of Veterinary Medicine and State Key Laboratory of Agrobiotechnology, China Agricultural University, Beijing, People's Republic of China; University of Minnesota, United States of America

## Abstract

Atypical porcine reproductive and respiratory syndrome (PRRS), which is caused by the Chinese highly pathogenic PRRS virus (HP-PRRSV), has resulted in large economic loss to the swine industry since its outbreak in 2006. However, to date, the region(s) within the viral genome that are related to the fatal virulence of HP-PRRSV remain unknown. In the present study, we generated a series of full-length infectious cDNA clones with swapped coding regions between the highly pathogenic RvJXwn and low pathogenic RvHB-1/3.9. Next, the *in vitro* and *in vivo* replication and pathogenicity for piglets of the rescued chimeric viruses were systematically analyzed and compared with their backbone viruses. First, we swapped the regions including the 5′UTR+ORF1a, ORF1b, and structural proteins (SPs)-coding region between the two viruses and demonstrated that the nonstructural protein-coding region, ORF1b, is directly related to the fatal virulence and increased replication efficiency of HP-PRRSV both *in vitro* and *in vivo*. Furthermore, we substituted the nonstructural protein (Nsp) 9-, Nsp10-, Nsp11- and Nsp12-coding regions separately; or Nsp9- and Nsp10-coding regions together; or Nsp9-, Nsp10- and Nsp11-coding regions simultaneously between the two viruses. Our results indicated that the HP-PRRSV Nsp9- and Nsp10-coding regions together are closely related to the replication efficiency *in vitro* and *in vivo* and are related to the increased pathogenicity and fatal virulence for piglets. Our findings suggest that Nsp9 and Nsp10 together contribute to the fatal virulence of HP-PRRSV emerging in China, helping to elucidate the pathogenesis of this virus.

## Author Summary

PRRS is a considerable threat to the pig industry worldwide. A large-scale atypical PRRS caused by highly pathogenic PRRSV (HP-PRRSV) that emerged in 2006 has resulted in considerable economic loss to Chinese pig production. The disease is characterized by a high body temperature (41°C–42°C), morbidity and by mortality of the affected pigs. Although the genomic marker, the 30-amino-acid deletion in its Nsp2-coding region has been previously verified to have no relation to its increased pathogenicity, the genomic region(s) associated with the fatal virulence of HP-PRRSV remain unclear. A series of chimeric viruses with swapped coding regions between HP- and LP-PRRSV were constructed, and their growth abilities and pathogenicities in piglets were analyzed. Our results demonstrated that Nsp9 and Nsp10 together contribute to the replication efficiency and the fatal virulence of HP-PRRSV for piglets. Our finding is not only the first unambiguous illumination concerning the key virulence determinant of Chinese HP-PRRSV but it also provides a novel insight for understanding the molecular pathogenesis of this virus and for designing new drugs and vaccines against PRRSV infection in the future.

## Introduction

Porcine reproductive and respiratory syndrome (PRRS) is characterized by reproductive failure in sows and respiratory diseases in all ages of pigs [1], [2]. This disease was first reported in the United States in the late 1980s [3], and in Germany in 1990, and then this disease became widespread throughout the world [4], [5]. The causative agent of this disease, PRRSV, was first identified in Europe in 1991 and independently in the USA in 1992 [6], [7]. PRRSV subsequently spread to the pig-producing countries and areas worldwide [1], [8]–[10], resulting in considerable economic loss to the pig industry worldwide [2], [11], [12].

PRRSV is an enveloped, positive-strand RNA virus that belongs to the family *Arteriviridae*, together with equine arteritis virus (EAV), mouse lactate dehydrogenase-elevating virus (LDV) and simian hemorrhagic fever virus (SHFV) [13]. Based on genetic and antigenic characteristics, two major genotypes of PRRSV, type 1 (European) and type 2 (North American), have been identified and share approximately 55–70% nucleotide identity [14]. The phylogenic analyses of the ORF7 sequence show that the European PRRSV can further be divided into three subtypes, pan European subtype 1 and East European subtypes 2 and 3 [15], and the North American PRRSV can be classified into at least 9 distinct genetic lineages [16].

The PRRSV genome is approximately 15 kb and consists of at least ten overlapping open reading frames (ORFs) [13], [17]–[20]. ORF1a and ORF1b, which occupy approximately three-fourths of the viral genome, encode the replicase polyproteins pp1a and pp1ab, which are autoproteolytically cleaved into at least 15 functional nonstructural proteins (Nsps) involved in virus replication and in transcription [21]–[26]. ORF1a encodes Nsp1α/β to Nsp8, including three important virus proteases, Nsp1 (papain-like cysteine protease), Nsp2 (chymotrypsin-like cysteine protease) and Nsp4 (3C-like serine protease). ORF1b is composed of Nsp9 (viral RNA-dependent RNA polymerase, RdRp), Nsp10 (RNA helicase), Nsp11 (endoribonuclease) and Nsp12. In particular, Nsp9 and Nsp10 are regarded as crucial enzymes for viral RNA sythesis [27]–[29]. Recently, Nsp11, with a NendoU domain, has been identified as an important participant in viral genome synthesis and in interferon (IFN) inhibition [30]–[33]. The ORFs 2 to 7 encode the structural proteins (SPs) of the virion, which are involved in virus infectivity, neutralizing antibody elicitation etc. [34]–[37].

The PRRSV strains circulating in the field are biologically, antigenically, and genetically heterogeneous, leading to extreme diversity in the clinical phenotypes and severities induced by the PRRSV infection [38]–[41]. Previous studies, based on genomic sequence comparisons between the parental virulent and attenuated strains, suggested that important determinants associated with virulence or attenuation might be scattered throughout the viral genome [42]–[47]. However, these speculations could not be easily proven, and some genomic mutations may result from the adaptation of the virus strain to propagate in the host cells but may not necessarily relate to *in vivo* virulence. A study swapped the 5′UTR+ORF1ab or SP+3′UTR region between a highly pathogenic strain MN184 and PRRS MLV and the pathogenicity analyses showed that both the regions from MLV could attenuate MN184, suggesting that the replicase gene is an important player in viral virulence and attenuation [48]. Another report indicated that PRRSV virulence was multigenic and that the Nsp3–8 regions were the major virulence determinants based on substituting a series of small genomic regions of a highly virulent strain FL-12 with their counterparts from an attenuated vaccine strain PrimePac [49].

An unparalleled, large-scale, atypical PRRS outbreak caused by the highly pathogenic PRRSV (HP-PRRSV) was documented in 2006 in China [40], [50], [51] and subsequently, in neighboring Asian countries. In contrast with previous Chinese PRRSV strains, the highly virulent PRRSV isolates with fatal pathogenicity for pigs can induce elevated body temperatures (over 41°C) and severe clinical presentations, including depression, anorexia, lethargy, rubefaction on the skin, respiratory distress, shivering and diarrhea [40], [50]. The highest mortality of nursery pigs in the infected farms could reach 100%. In recent years, HP-PRRSV has been recognized as the dominating virus in China [52]. Chinese HP-PRRSV strains share a unique genomic characteristic, namely a discontinued 30-amino-acid (30-aa) deletion within their Nsp2-coding regions [52]–[54]. Our previous studies have confirmed that this deletion is not related to the fatal virulence of HP-PRRSV [40]; however, whether the genome of the virus has the fatal virulence-determining or related region(s) remains unknown. A strain belonging to the 3 subtypes of PRRSV genotype 1, Lena, with increased virulence, occurred in Eastern Europe, and a recent study demonstrated that Lena, with high replication efficiency, caused high fevers, anorexia, depression and severe respiratory problems, similar to Chinese HP-PRRSV [55], [56]. Thus, analyzing the molecular mechanism associated with the enhanced virulence of PRRSV is helpful for controlling this disease.

The objective of this study was to explore the genomic region(s) possibly related to the virulence of Chinese HP-PRRSV. We used the infectious clones (pWSK-JXwn and pWSK-HB-1/3.9) of a HP-PRRSV strain, JXwn06, and of a low pathogenic PRRSV (LP-PRRSV) but genetically close strain, HB-1/3.9, as backbones and constructed a series of chimeric clones by individually exchanging the corresponding coding region within the genome between the two parental clones. Next, we rescued the viruses, analyzed their growth efficiency *in vitro* and *in vivo*, as well as their pathogenicities for piglets, and, finally determined the genomic regions that contribute to the fatal virulence of Chinese HP-PRRSV.

## Materials and Methods

### Ethics statement

The animal trials in this study were performed according to the Chinese Regulations of Laboratory Animals—*The Guidelines for the Care of Laboratory Animals* (Ministry of Science and Technology of People's Republic of China) and *Laboratory Animal-Requirements of Environment and Housing Facilities* (GB 14925-2010, National Laboratory Animal Standardization Technical Committee). The license number associated with their research protocol was 20120611-01, which was approved by The Laboratory Animal Ethical Committee of China Agricultural University.

### Cells and viruses

MARC-145 cells and BHK-21 cells were cultured in Gibco Dulbecco's modified Eagle medium (DMEM) (Invitrogen), which was supplemented with 10% fetal bovine serum (FBS) (HyClone) at 37°C under a humid 5% CO_2_ atmosphere. BHK-21 cells were used for full-length infectious cDNA clone transfection. Pulmonary alveolar macrophages (PAMs), which are the primarily target cells for PRRSV, were prepared from the lung lavage fluid of 5- to 6-week-old healthy piglets free of PRRSV as previously described [6], [57]. Primary PAMs were maintained in GIBCOTM RPMI 1640 medium (Invitrogen), which was supplemented with 10% FBS, 100 mg/ml kanamycin, 50 U/ml penicillin, 50 mg/ml streptomycin, 25 mg/ml polymixin B and 1 mg/ml fungizone at 37°C, 5% CO_2_ or were cryopreserved in liquid nitrogen for later use. The full-length infectious cDNA clone plasmids (pWSK-JXwn and pWSK-HB-1/3.9) of HP-PRRSV JXwn06 and of low pathogenic (LP) PRRSV HB-1/3.9 and the rescued viruses (RvJXwn and RvHB-1/3.9) were used in this study [40], [58].

### Construction of chimeric full-length cDNA clones

**Figure 1:**
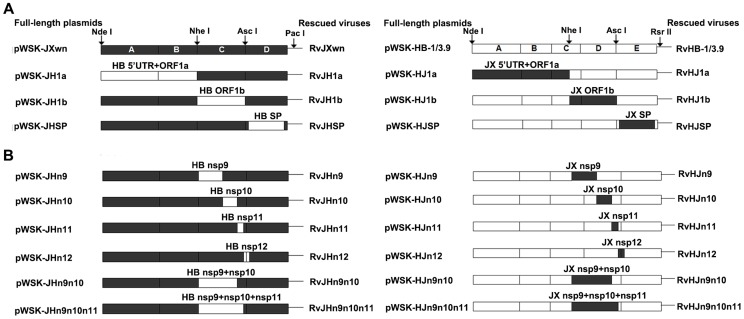
Construction strategy of the full-length cDNA clones. (A) Full-length infectious clones with exchanged 5′UTR+ORF1a, ORF1b, and structural proteins (SPs)-coding regions. (B) Full-length infectious clones with exchanged nonstructural protein (Nsp)-coding region within ORF1b. The boxes represent the genomic fragments of parental backbone viruses RvJXwn (black) or RvHB-1/3.9 (white). Unique restriction enzyme sites used for cloning are shown above the bars. The designations of each full-length plasmid are shown on the left side and each rescued virus on the right side.

The 5′UTR+ORF1a or ORF1b between pWSK-JXwn and pWSK-HB-1/3.9 was swapped using the unique restriction enzymes (New England Biolabs) *Nde*I and *Nhe*I or *Nhe*I and *Asc*I, respectively. The structural protein (SP)-coding regions from one full-length plasmid and flanking segments of corresponding sites from the other full-length plasmid were individually amplified by PCR using the primers listed in Table S1. Then, a new fragment D of pWSK-JXwn, which contained the SP-coding region of pWSK-HB-1/3.9, and a new fragment E of pWSK-HB-1/3.9, which contained the SP-coding region of pWSK-JXwn were generated using fusion PCR (Fig. 1A). Then, the PCR products were purified, digested by the restriction enzymes (*Asc*I and *Pac*I for fragment D, *Asc*I and *Rsr*II for fragment E), and finally inserted back into the backbones, which were digested by the same restriction enzymes. The chimeric full-length cDNA clones with the pWSK-JXwn backbone were named pWSK-JH1a, pWSK-JH1b and pWSK-JHSP; correspondingly, the chimeric clones with the pWSK-HB-1/3.9 backbone were designated pWSK-HJ1a, pWSK-HJ1b and pWSK-HJSP.

The strategies of exchanging the nonstructural protein (Nsp)-coding regions were similar to the method described above. The target region was amplified from one full-length plasmid and linked with the flanking fragments amplified from the other full-length plasmid by fusion PCR using the primers in Table S1, namely, a series of new fragments, C and D, of pWSK-JXwn containing the Nsp9-, Nsp10-, Nsp11-, Nsp12-, Nsp9- and Nsp10- or Nsp9-, Nsp10- and Nsp11-coding regions of pWSK-HB-1/3.9. Additionally, a series of new fragments, D and E, of pWSK-HB-1/3.9 containing the corresponding regions of pWSK-JXwn were constructed. Then, the fusion PCR products were inserted into the full-length backbone plasmid by using the closest unique enzyme sites (Fig. 1B). The chimeric full-length cDNA clones with the pWSK-JXwn backbone, containing the Nsp-coding region from pWSK-HB-1/3.9, were individually designated pWSK-JHn9, pWSK-JHn10, pWSK-JHn11, pWSK-JHn12, pWSK-JHn9n10 and pWSK-JHn9n10n11, and correspondingly, the chimeric clones with the RvHB-1/3.9 backbone were designated pWSK-HJn9, pWSK-HJn10, pWSK-HJn11, pWSK-HJn12, pWSK-HJn9n10 and pWSK-HJn9n10n11.

### Recovery of chimeric viruses

To rescue the chimeric viruses, each full-length cDNA clone plasmid was separately linearized using the restriction enzyme *Pac*I (for cDNA clones with RvJXwn as the backbone) or *Rsr*II (for cDNA clones with RvHB-1/3.9 as the backbone). Then, the purified full-length cDNA clone was transcribed and capped using a mMessage high-yield capped RNA transcription kit (Ambion) according to the manufacturer's protocol. The purified RNAs were transfected into BHK-21 cells as previously described [40]. At 24 h post-transfection, the supernatants were harvested and serially passaged for three times in MARC-145 cells. The MARC-145 cells infected with the rescued viruses were examined by indirect immunofluorescence assay (IFA) using the monoclonal antibody (McAb) SDOW17 (Rural Technologies), which is specific for the PRRSV N protein [59], [60]. To further verify the correctness of the exchanged regions, the RNAs of third-passage chimeric viruses were extracted and subjected to RT-PCR, followed by sequencing.

### The growth kinetics of the rescued viruses in MARC-145 cells and in primary PAMs

To analyze the *in vitro* growth characteristics, the MARC-145 cell monolayers or primary PAMs in T-25 flasks were individually infected with each chimeric virus and with their parental viruses at the same multiplicity of infection (MOI) of 0.01. The titers of virus in the supernatants at different time points were determined using a microtitration infectivity assay and expressed as 50% tissue culture infective dose per ml (TCID_50_/ml). Briefly, the confluent monolayers of MARC-145 cells cultured in 96-well plates were incubated with 10-fold serially diluted virus suspensions. After absorption for 1 h at 37°C, the supernatants were removed, and 5% DMEM was added. The plates were incubated for an additional 48 to 60 h, and then the virus titers were determined. Each time point was independently repeated three times.

### Animal trials for pathogenicity analyses of the rescued chimeric viruses

Healthy, 6-week-old, landrace piglets that were free of PRRSV, classic swine fever virus (CSFV), pseudorabies virus (PRV), porcine circovirus type 2 (PCV2) and *M. hyopneumoniae* infections were obtained from Beijing Center for SPF Swine Breeding & Management. All piglets were confirmed to be negative for PRRSV and PCV2 antibodies, as determined using commercial ELISA kits and using RT-PCR or PCR for viral nucleic acid detection in sera. The animals were raised in the animal facilities at China Agricultural University (CAU). For the first batch of animal trials, forty-five piglets were randomly divided into nine groups. The animals in each group (*n* = 5) were separately raised in different isolation rooms. Each piglet in each infection group was intranasally administered with 2 ml of each virus containing 2×10^5^ TCID_50_ (RvJXwn, RvJH1a, RvJH1b, RvJHSP, RvHB-1/3.9, RvHJ1a, RvHJ1b or RvHJSP). Each piglet in the control group was mock-inoculated with the same dose of MARC-145 cell culture supernatant. For the second batch of animal trials, fifty-five piglets were randomly allotted to eleven groups (*n* = 5). Each piglet in each infection group was intranasally inoculated with 2 ml of each virus containing 2×10^5^ TCID_50_ (RvJHn9, RvJHn10, RvJHn9n10, RvJHn9n10n11, RvHJn9, RvHJn10, RvHJn9n10 or RvHJn9n10n11). The piglets in the control group were mock inoculated with 2 ml of MARC-145 cell culture supernatant. All the survived piglets were euthanized and necropsied on day 21 post-inoculation (pi).

### Clinical examinations

The daily observation of clinical signs and measurement of rectal temperatures were performed throughout the experiments. For the second batch of animal experiments, the clinical symptoms of each piglet were monitored and scored. A detailed scoring system is summarized in Table S2. Meanwhile the piglets were weighed on days 0, 7, 14 and 21 pi to calculate the average daily gain (ADG).

### Necropsy and gross pathology examinations

Necropsy and gross pathological examinations of lungs were immediately performed once the piglets died during the experiment, or the survived piglets were euthanized at the termination of the experiment. The total amount of lungs affected by grossly visible pneumonia were evaluated as the percentage of lesions noted per lobe, and an overall level of gross pathology was determined according to the previously described standard scoring system [61].

### Microscopic pathological changes and immunohistochemistry (IHC) examinations

Lung tissues were collected, fixed with 4% paraformaldehyde solution at room temperature for 48 h and then processed by routine histopathological procedures. Each sample was examined on two sections. One section was stained with hematoxylin and eosin (H&E) for observing pathological changes, and the other section was stained with McAb SDOW17 at 1∶2000 dilutions for detecting PRRSV N antigen positive cells.

Lung sections stained with H&E were blindly evaluated by a veterinary pathologist and the scores from 0 to 4, which accounted for the distribution and severity of interstitial pneumonia were recorded as previously described [61]. The indications for the scores were as follows: 0 = no microscopic lesions; 1 = mild, focal to multifocal interstitial pneumonia; 2 = moderate, multifocal to coalescing interstitial pneumonia; 3 = severe, patchy to coalescing and extensive interstitial pneumonia; and 4 = severe and diffuse interstitial pneumonia. The detection of the PRRSV antigen was executed through a ranked score of 0–4, which was used to evaluate of the number of positive cells per section taken from each block, as previously described [62]. The indications for the scores were as follows: 0 = no positive cells, 1 = 1–10 positive cells, 2 = 11–30 positive cells, 3 = 31–100 positive cells, and 4 =  or >100 positive cells.

### Viremia and serology analysis

Serum samples were collected on days 0, 3, 5, 7, 10, 14 and 21 pi to examine the viral loads of the infected animals using a microtitration infectivity assay, as described above, and to detect PRRSV-specific antibodies using a commercial IDEXX Herdchek PRRS 2XR ELISA kit.

### Statistical analysis

The significant differences of the *in vitro* experiment and animal trials were analyzed using a one-way or two-way RM ANOVA in the GraphPad Prism (version 5.0) software. A *t-*test was used to estimate the variability among the gross lesion, histopathological and immunohistochemistry scores of lungs. Differences were considered statistically significant at a *P* value of <0.05 and extremely significant at a value of *P*<0.01 or *P*<0.001.

### Accession numbers

GenBank accession numbers (http://www.ncbi.nlm.nih.gov/Genbank):

PRRSV JXwn06, complete genome: EF641008

PRRSV HB-1/3.9, complete genome: EU360130

## Results

### Recovery and *in vitro* growth properties of the chimeric viruses with exchanged 5′UTR+ORF1a, ORF1b or structural proteins-coding regions

Six chimeric viruses were successfully rescued from the chimeric infectious clones constructed by swapping 5′UTR+ORF1a, ORF1b or structural proteins (SP)-coding regions between pWSK-JXwn and pWSK-HB-1/3.9 plasmids. The MARC-145 cells infected with each chimeric virus were shown to be positive for PRRSV N protein using IFA (Supplemental Fig. S1). Further sequencing of the replaced regions and their flanking areas of the third-passage viruses confirmed that the substitutions were consistent with the original design and that no additional mutations were introduced during the construction (data not shown). The six rescued viruses were individually designated RvJH1a, RvJH1b, RvJHSP, RvHJ1a, RvHJ1b and RvHJSP (Fig. 1A).

**Figure 2:**
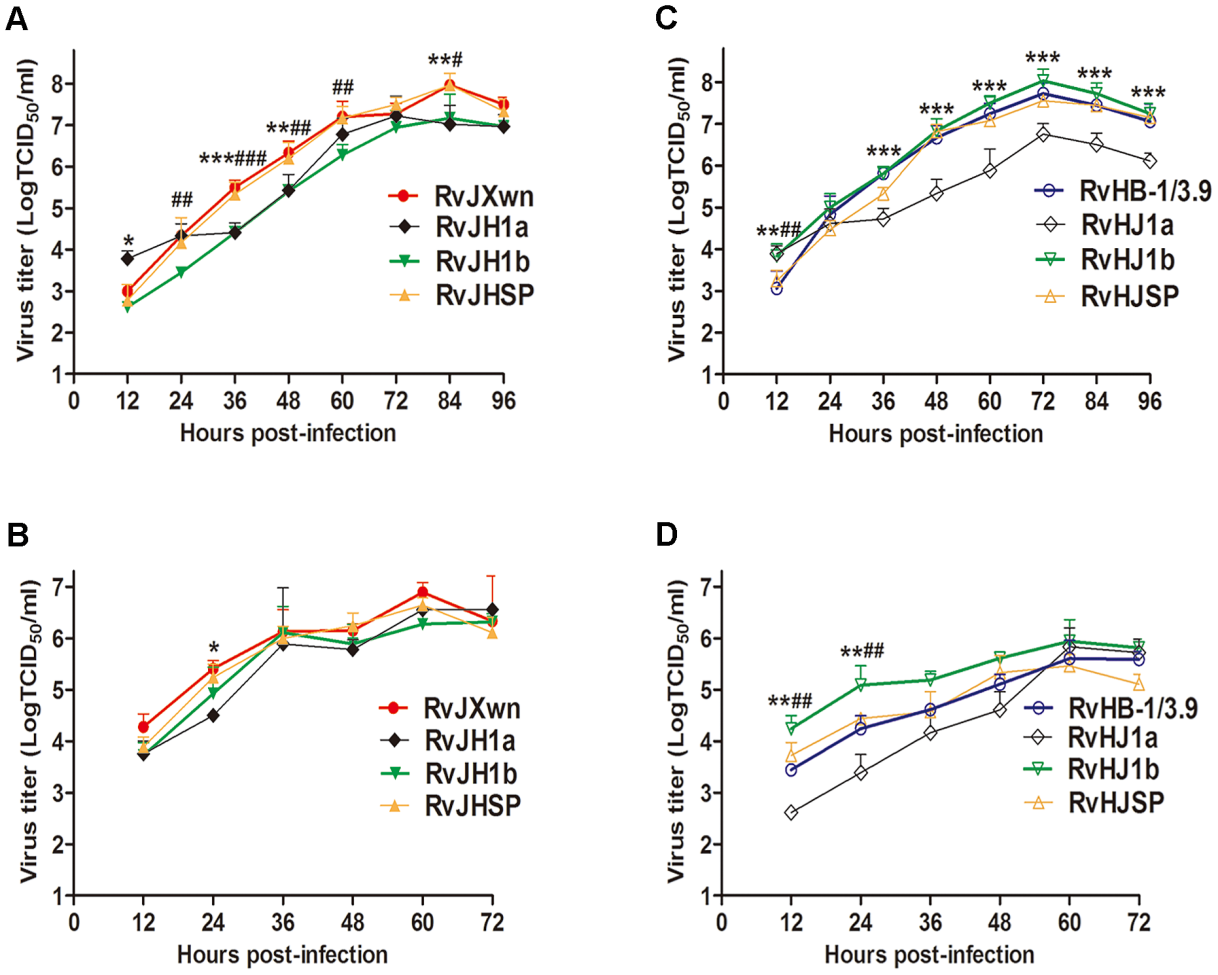
The growth kinetics of the rescued viruses with the exchanged 5′UTR+ORF1a, ORF1b and SP-coding regions. (A) and (C) in MARC-145 cells; (B) and (D) in primary PAMs. Asterisk indicates a significant difference in the virus titers between RvJXwn (red) and RvJH1a (black) or between RvHB-1/3.9 (blue) and RvHJ1a (black) (*P<0.05; **P<0.01; ***P<0.001). Pound (#) indicates a significant difference between RvJXwn and RvJH1b (green) or between RvHB-1/3.9 and RvHJ1b (green) (#*P*<0.05; ##*P*<0.01; ###*P*<0.001). RvJHSP and RvHJSP (yellow).

The growth kinetics of chimeras in parallel with their parental viruses, RvJXwn and RvHB-1/3.9, were evaluated by infecting MARC-145 cells or primary PAMs. The results showed that RvJH1a and RvHJ1a had lower growth kinetics, whereas RvJHSP and RvHJSP had similar growth kinetics to their parental backbone viruses in both MARC-145 cells and primary PAMs (Fig. 2), indicating that exchanging the 5′UTR+ORF1a region can impair the replication efficiency of the viruses, whereas substituting the SP-coding region does not affect this efficiency. Importantly, compared with the backbone viruses, RvJH1b showed a reduced replication efficiency in both MARC-145 cells and primary PAMs (Fig. 2A and B), particularly in MARC-145 cells; in contrast, RvHJ1b exhibited an enhanced replication efficiency with obviously higher titers at all the time points, particularly in primary PAMs (Fig. 2C and D), suggesting that the ORF1b of HP-PRRSV is related to its growth efficiency *in vitro*.

### ORF1b affects the fatal virulence of HP-PRRSV for piglets

To further determine the effect of the exchanged genomic regions on the pathogenicity of PRRSV, animal inoculation experiments were performed with 6-week-old piglets and the pathogenicities of the chimeric viruses were compared with their parental backbone viruses, RvJXwn and RvHB-1/3.9. The observation of clinical symptoms showed that only one piglet in RvJH1a-inoculated group had dyspnoea, and the piglets in the RvHJ1a-inoculated group had no obvious clinical symptoms, similar to the RvHB-1/3.9-infected group. In contrast, the piglets infected with RvJHSP developed marked clinical symptoms, similar to those in the RvJXwn-inoculated group, including depression, anorexia, lethargy, rubefaction on the skin and in the ears, respiratory distress and shivering; whereas the animals in the RvHJSP-inoculated group presented some moderate signs until the end of experiment. The piglets in the RvJH1b-inoculated group only showed mild coughing and sneezing, and conversely, the piglets inoculated with RvHJ1b exhibited severe respiratory conditions, similar to the RvJXwn-inoculated group, indicating that the substitution of RvJXwn ORF1b by that of RvHB-1/3.9 could remarkably reduce the viral pathogenicity and that the replacement of RvHB-1/3.9 ORF1b by that of RvJXwn could enhance the viral pathogenicity. No clinical signs were observed in the control group during the experiment period.

**Figure 3:**
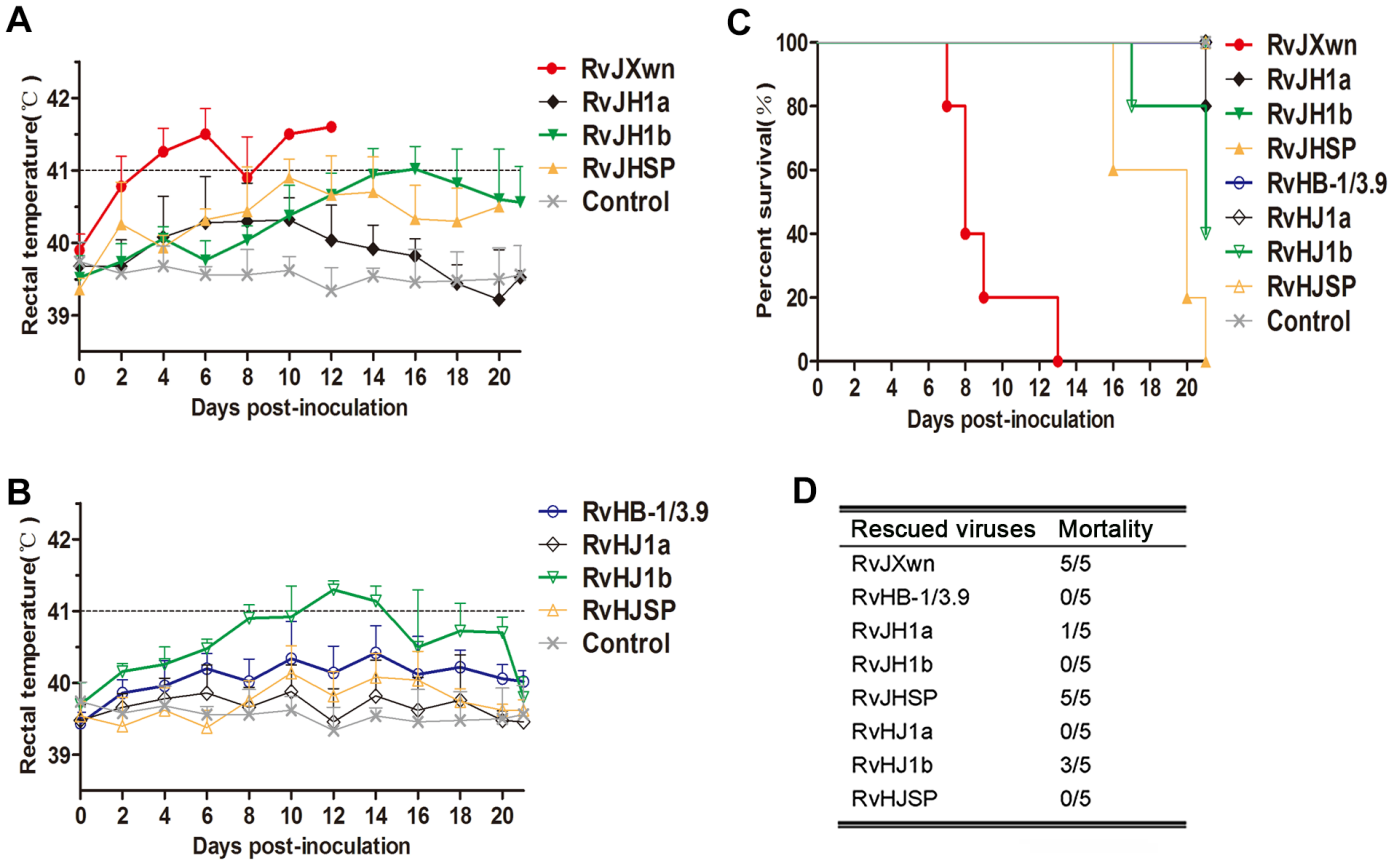
The rectal temperatures and the mortality of piglets inoculated with the rescued virus with the exchanged coding regions. Body temperatures of piglets inoculated with rescued viruses with the RvJXwn backbone (A) and with the RvHB-1/3.9 backbone (B). Body temperatures are shown as the means ± standard deviations (error bars), except the number of survival piglets in each group was less than two. The survival curves (C) and mortalities (D) of infected piglets in each group (*n* = 5) are shown.

Rectal temperature measurements indicated that both the RvJH1a- or RvHJ1a-inoculated piglets had lower temperatures than the parental backbone virus groups; in contrast, the piglets inoculated with RvJHSP had a peak temperature (nearly 41°C) on day 10 pi and then hovered approximately 40.5°C (Fig. 3A and B). The RvHJSP-inoculated group had similar temperatures to the RvHB-1/3.9-inoculated group. Nevertheless, the rectal temperatures of the RvJH1b-inoculated piglets were obviously lower than those temperatures of the RvJXwn-inoculated group with a high fever during the experiment, except for a temperature of 41°C on day 16 pi; in contrast, the rectal temperatures of piglets inoculated with RvHJ1b elevated at 24 h pi with a temperature of over 41°C for couple days compared with the RvHB-1/3.9 group (Fig. 3A and B).

The mortalities of the inoculated animals were recorded. As shown in Fig. 3C and D, only one piglet died in the RvJH1a-inoculated group, and all animals in RvHJ1a- and RvHB-1/3.9-inoculated groups survived. However, all piglets in the RvJHSP- and RvJXwn-inoculated groups died, whereas all piglets survived in the RvHJSP-inoculated group during the experiment period. Most importantly, all piglets survived in the RvJH1b-inoculated group, whereas three piglets died in the RvHJ1b-inoculated group within day 17 to 21 pi, showing that RvJH1b has a remarkably reduced virulence, while RvHJ1b exhibits obviously an increased mortality for piglets.

**Figure 4:**
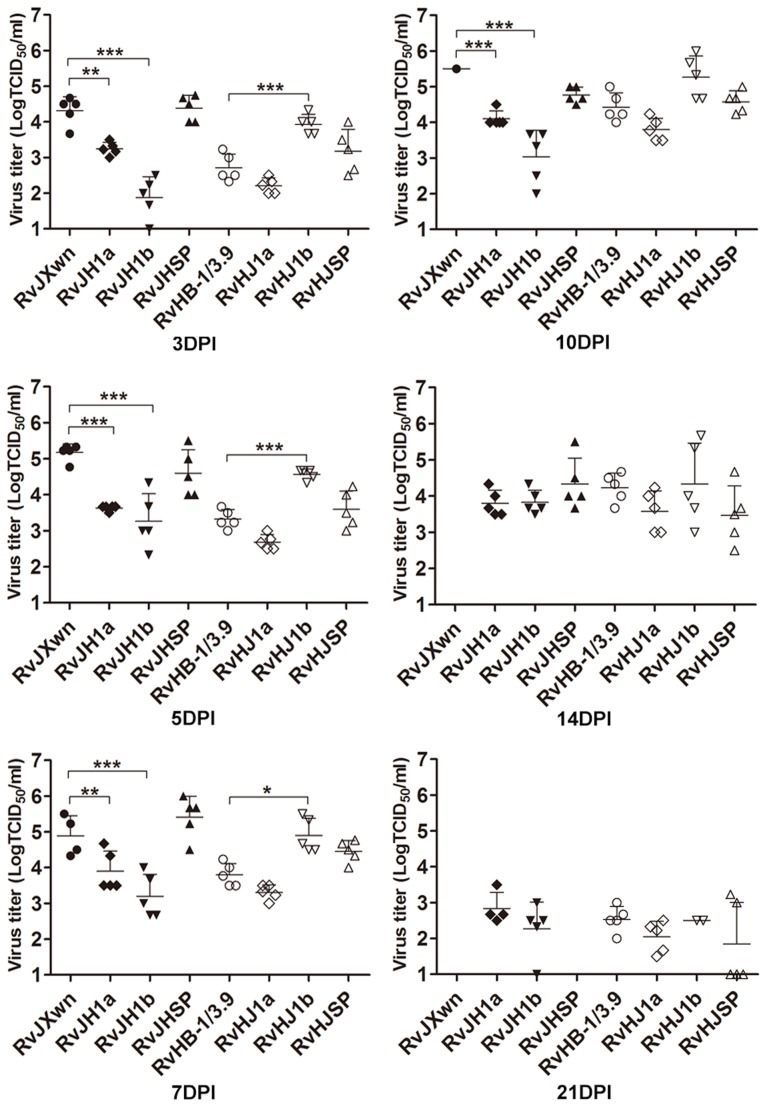
Viral loads in sera of piglets inoculated with the rescued viruses with the exchanged coding regions. Virus titers were determined by a microtitration infectivity assay. The data are shown as the means ± standard deviations (error bars), except only one piglet in RvJXwn-infected group survived on day 10 pi. Asterisk indicates significant differences in viral loads between the chimeric virus and RvJXwn or RvHB-1/3.9 (*P<0.05; **P<0.01; ***P<0.001).

The virus loads in the sera of infected piglets were assayed using a microtitration infectivity assay (Fig. 4). The data showed that both RvJH1a- and RvHJ1a-inoculated piglets had lower virus loads than their parental virus groups, whereas no obvious difference of virus titers in sera was found between the RvJHSP- or RvHJSP-infected group and its parental virus group. Interestingly, the virus titers in the sera of RvJH1b-inoculated group were significantly lower than those titers of RvJXwn-inoculated group from day 3 to 10 pi (*P*<0.001); in contrast, the virus titers in the sera of RvHJ1b-inoculated group were statistically higher than those titers of RvHB-1/3.9-inoculated group on days 3, 5, and 7 pi (*P*<0.05, *P*<0.001).

Collectively, our above results clearly demonstrate that ORF1b affects the fatal virulence of HP-PRRSV.

### Nsp9 and Nsp10 together were closely related to the replication efficiency of HP-PRRSV *in vitro*

To further determine which coding region(s) within the ORF1b play crucial roles in the increased virulence of HP-PRRSV, we swapped Nsp9, Nsp10, Nsp11 and Nsp12; or Nsp9 and Nsp10 simultaneously; or Nsp9, Nsp10 and Nsp11 simultaneously between the infectious cDNA clones of RvHB-1/3.9 and RvJXwn and successfully rescued twelve chimeric viruses. The MARC-145 cells infected with each chimeric virus were shown to be positive for PRRSV N protein using IFA (Supplemental Fig. S2). Sequencing analyses were also performed to ensure the correctness of the substitutions (data not shown). These rescued viruses were individually designated RvJHn9, RvJHn10, RvJHn11, RvJHn12, RvJHn9n10, RvJHn9n10n11, RvHJn9, RvHJn10, RvHJn11, RvHJn12, RvHJn9n10 and RvHJn9n10n11.

**Figure 5:**
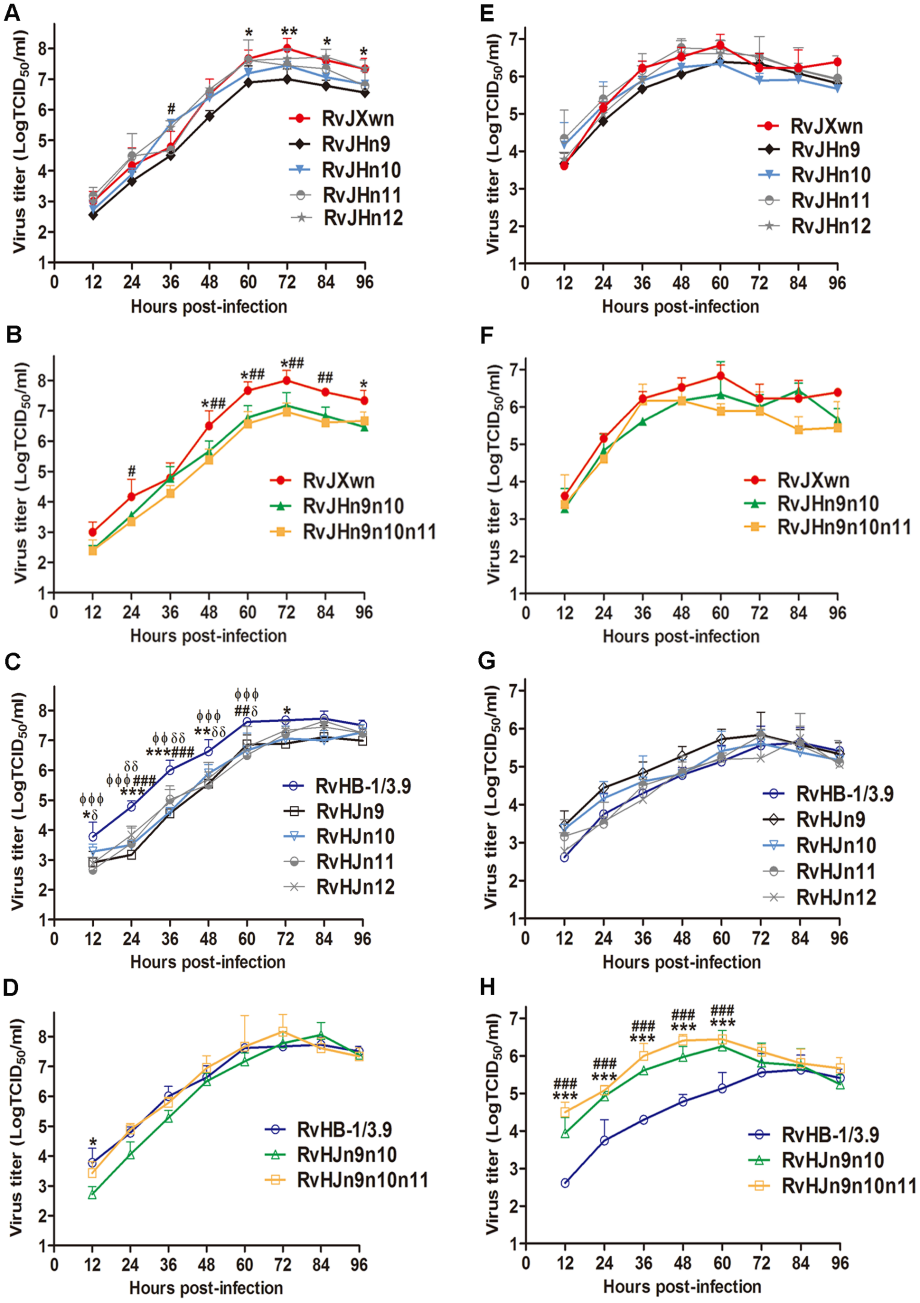
Growth kinetics of the rescued viruses with the exchanged Nsp-coding region within ORF1b. The growth curves of each chimeric virus, RvJXwn and RvHB-1/3.9 in MARC-145 cells (A, B, C and D) and in primary PAMs (E, F, G and H) are shown. Virus titers from 12 h to 96 h pi were determined by microtitration infectivity assays. The data are shown as the means ± standard deviations (error bars) from three independent trials. Asterisk (*) indicates a significant difference in virus titers between RvJXwn (red) and RvJHn9 (black), or RvJHn9n10 (green), or between RvHB-1/3.9 (blue) and RvHJn9 (black) or RvHJn9n10 (green) (*P<0.05; **P<0.01; ***P<0.001). Pound (#) indicates a significant difference between RvJXwn and RvJHn10 (light blue) or RvJHn9n10n11 (yellow), or between RvHB-1/3.9 and RvHJn10 (light blue) or RvHJn9n10n11 (yellow) (##*P*<0.01; ###*P*<0.001). Phi (Φ) indicates a significant difference between RvHB-1/3.9 and RvHJn11 (gray) (ΦΦ*P*<0.01; ΦΦΦ*P*<0.001). Delta (δ) indicates a significant difference between RvHB-1/3.9 and RvHJn12 (gray) (δ*P*<0.05; δδ*P*<0.01).

To characterize and to compare the growth properties between these chimeras and their parental viruses, their growth kinetics were analyzed by infecting MARC-145 cells or primary PAMs with each virus. In MARC-145 cells, as shown in Fig. 5A, RvJHn9 showed obviously lower virus titers than RvJXwn, with significant differences from 60 h to 96 h pi (*P*<0.05, *P*<0.01). Likewise, RvJHn10 had lower virus titers than RvJXwn; however, RvJHn11 and RvJHn12 showed similar growth patterns to their parental virus RvJXwn. In contrast, with an individual Nsp-coding region substitution on the RvJXwn backbone, RvJHn9 had a slower growth rate than the other three chimeric viruses. Moreover, both RvJHn9n10 and RvJHn9n10n11 displayed significantly lower virus titers than RvJXwn at some time points (*P*<0.05, *P*<0.01) (Fig. 5B). As shown in Fig. 5C, compared with the parental virus RvHB-1/3.9, all four rescued viruses with single region substitution showed significantly lower virus titers at many time points (*P*<0.05, *P*<0.01, *P*<0.001), whereas RvHJn9n10 had slightly lower virus titers than RvHB-1/3.9. Additionally, RvHJn9n10n11 had slightly higher virus yields than RvHB-1/3.9 at some time points. Moreover, the peak titers of RvHJn9n10 and RvHJn9n10n11 were both higher than that of RvHB-1/3.9, without a significant difference (Fig. 5D).

In primary PAMs, the growth kinetics revealed that RvJHn9 and RvJHn10 had lower virus titers than RvJXwn at the majority of time points without significant differences, whereas both RvJHn11 and RvJHn12 presented a similar growth curve to that of RvJXwn. The data also showed that RvJHn9 exhibited a lower virus yield than RvJHn11 and RvJHn12 for all time points and than RvJHn10 within 12 h to 60 h pi (Fig. 5E). Furthermore, compared with RvJXwn, RvJHn9n10 had a lower replication efficiency with a peak titer delayed by 24-h. Similarly, RvJHn9n10n11 showed obviously lower virus titers than RvJXwn at some time points (Fig. 5F). In contrast, RvHJn9 and RvHJn10 showed higher virus titers than RvHB-1/3.9, without significant differences, whereas both RvHJn11 and RvHJn12 had a similar growth curve to that of RvHB-1/3.9 (Fig. 5G). Among the four chimeric viruses with the backbone of RvHB-1/3.9, RvHJn9 displayed a higher virus yield. As shown in Fig. 5H, both RvHJn9n10 and RvHJn9n10n11 had obviously higher virus titers than RvHB-1/3.9, with significant differences from 12 h to 60 h pi (*P*<0.01, *P*<0.001); and moreover their peak titers were 10 times higher than that of RvHB-1/3.9.

Our above results demonstrated that the substitution of the Nsp9- and/or Nsp10-coding region(s) could obviously affect the virus replication efficiency *in vitro*, particularly when exchanging both of these regions together, whereas the replacement of the Nsp11- or Nsp12-coding region did not have this effect, indicating that Nsp9 and Nsp10 together are closely related to the replication efficiency of HP-PRRSV *in vitro*.

### Nsp9 and Nsp10 together contribute to the fatal virulence of HP-PRRSV for piglets

To further analyze whether the Nsp9- and Nsp10-coding region alone or together is related to the increased pathogenicity of HP-PRRSV *in vivo*, the pathogenicities of the chimeric viruses RvJHn9, RvJHn10, RvJHn9n10, RvJHn9n10n11, RvHJn9, RvHJn10, RvHJn9n10 and RvHJn9n10n11 in piglets were investigated and compared with their parental viruses.

**Figure 6:**
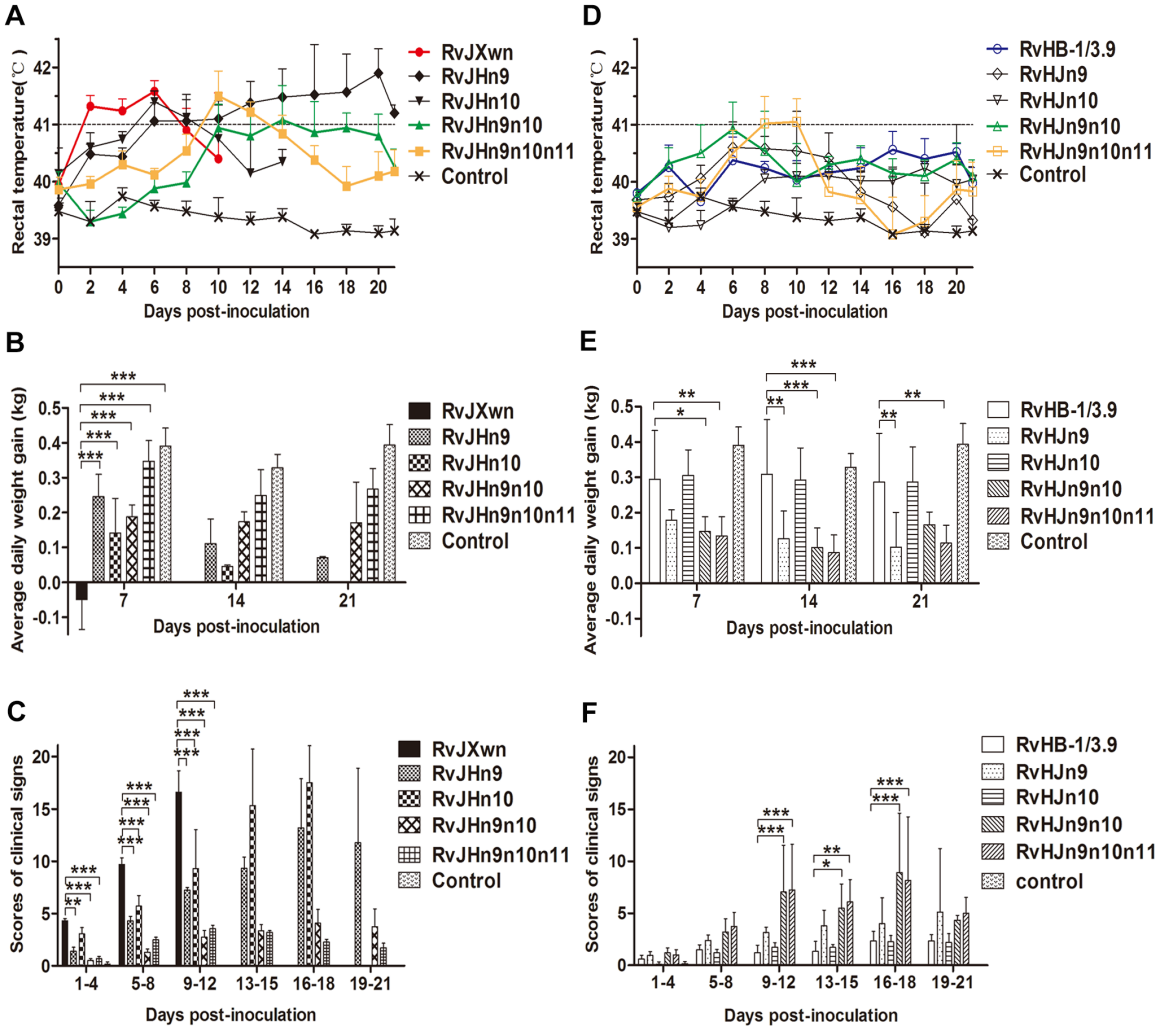
The rectal temperatures, clinical scores and average daily gains of piglets inoculated with the rescued viruses. The body temperatures, average clinical scores and average daily gains (ADG) of piglets inoculated with the rescued viruses with the RvJXwn backbone (A, C and E) or with the RvHB-1/3.9 backbone (B, D and F) are shown. The data are shown as the means ± standard deviations (error bars). The clinical scoring included the gross clinical score (GCS), respiratory clinical score (RCS) and nervous signs score (NSS). Total scores for each piglet represented the sum of the GCS, RCS and NSS. An additional five score was calculated in the total scores when the piglet died. Each piglet was scored from 0–20, and the mean values of day 1 to 4 pi, 5 to 8 pi, 9 to 12 pi, 13 to 15 pi, 16 to 18 pi and 19 to 21 pi were calculated. Asterisk indicates a significant difference between the chimeric virus and its parental backbone virus, RvJXwn or RvHB-1/3.9 (*P<0.05; **P<0.01; ***P<0.001).

The animals were inoculated with each chimeric virus. Rectal temperatures of the inoculated piglets were daily measured. As shown in Fig. 6A, the RvJHn9-infected piglets had a rising body temperature, with an average of 41°C on day 6 pi, whereas the body temperatures of the RvJHn10-infected group began to rise on day 1 pi, and then reached a peak of 41.4°C, similar to those temperatures of RvJXwn-infected group with an average of 41°C on day 2 pi. However, compared with the RvJXwn-infected group, the temperatures of the RvJHn9n10-infected group slowly elevated to a peak of 41.1°C on day 14 pi, with a extreme delay and then gradually declined. Similarly, the RvJHn9n10n11-infected group slowly reached its peak on day 10 pi and then rapidly decreased. In contrast, the temperatures of the RvHJn9-infected group started to rise earlier after day 2 pi, and reached over 40°C from day 4 to 12 pi, whereas the RvHJn10-infected group had a slightly lower temperature, compared with the RvHB-1/3.9-infected group. Importantly, the temperatures of RvHJn9n10-infected group reached over 40°C on day 2 pi, then elevated quickly to a peak of approximately 41°C on day 6 pi, and maintained beyond 40°C, with a significantly greater rising tendency, compared with the RvHB-1/3.9-, RvHJn9- or RvHJn10-infected groups during the first half of the experiment. The RvHJn9n10n11-infected group also exhibited higher temperatures with over 41°C from day 8 to 10 pi (Fig. 6B). No body temperature reaction was observed in the control group during the experiment period.

The clinical signs of the infected piglets were observed and scored. Compared with the RvJXwn-infected group, the RvJHn9-infected piglets showed moderate and slower progress of disease. All piglets in the RvJHn10-infected group exhibited similar severe signs to the RvJXwn-infected group, whereas the piglets in both RvJHn9n10- and RvJHn9n10n11-infected groups only displayed transient fever and mild respiratory symptoms with significantly lower clinical symptom scores than the RvJXwn- (*P*<0.001), RvJHn9- or RvJHn10-infected group (Fig. 6C). In contrast, in the RvHJn9-infected group, one piglet died with severe symptom from day 15 pi onward, and others showed moderate anorexia, sneezing and coughing. No obvious clinical signs were observed in the RvHJn10-infected group, similar to the RvHB-1/3.9-infected group with occasional sneezing and coughing. In contrast, the piglets in RvHJn9n10- and RvHJn9n10n11-infected groups showed typical symptoms caused by RvJXwn from day 6 pi onwards, with higher clinical symptom scores than the RvHB-1/3.9-, the RvHJn9- or RvHJn10-infected group (Fig. 6D). No abnormal clinical symptoms were observed in the control group during the entire experiment.

The body weights of the infected and control piglets were recorded weekly to calculate the average daily gain (ADG). As shown in Fig. 6E, the RvJXwn-, RvJHn9- and RvJHn10-infected groups had significantly lower ADG. In contrast, the RvJHn9n10-infected group had an ADG of approximately 0.17 kg, which was significantly higher than that of RvJXwn-, RvJHn9- or RvJHn10-infected group (*P*<0.05, *P*<0.01, *P*<0.001). The RvJHn9n10n11-infected group showed a higher ADG of nearly 0.26 kg, with significant differences in comparison with other three groups (*P*<0.05, *P*<0.01, *P*<0.001). As shown in Fig. 6F, the RvHJn9-infected group had a lower ADG than RvHB-1/3.9-infected group, whereas the RvHJn10-infected group had an ADG of approximately 0.30 kg, similar to the RvHB-1/3.9-infected group. Moreover, the RvHJn9n10- and RvHJn9n10n11-infected groups showed a remarkably lower ADG compared with RvHB-1/3.9- or RvJHn10-infected group. The ADG of the control group was statistically higher than those ADG values of all infected groups (*P*<0.001), except for RvHB-1/3.9 and RvHJn10 groups.

**Figure 7:**
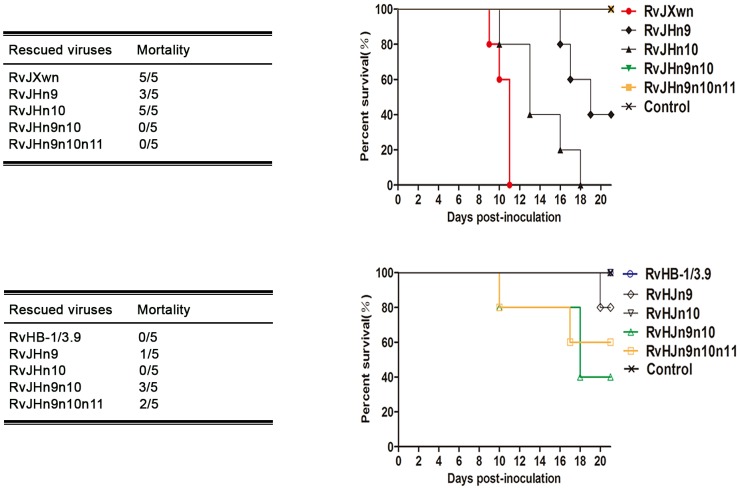
The mortality and survival curve of piglets inoculated with the rescued viruses. The mortalities and survival curves of piglets infected with rescued viruses in each group are shown (*n* = 5).

The deaths of the animals in each group were recorded. As shown in Fig. 7, all piglets in the RvJXwn- or RvJHn10-infected group died, although the survival time of the RvJHn10 group was obviously prolonged. Three piglets in the RvJHn9-infected group died, whereas all piglets inoculated with RvJHn9n10 or with RvJHn9n10n11 survived during the experiment. Additionally, no piglets died in the RvHB-1/3.9- and RvHJn10-infected groups, whereas one piglet in the RvHJn9-infected group died on day 20 pi. Importantly, three piglets in the RvHJn9n10-infected group died during day 10 to 18 pi, and two in the RvHJn9n10n11-infected group died on days 10 and 17 pi, respectively.

Taken together, these results clearly showed that simultaneously replacing the Nsp9- and Nsp10-coding regions of RvJXwn by RvHB-1/3.9 could remarkably reduce its fatal virulence for piglets, and in contrast, replacing of both the Nsp9- and Nsp10-coding regions of RvHB-1/3.9 by RvJXwn could significantly enhance its virulence for piglets.

**Figure 8:**
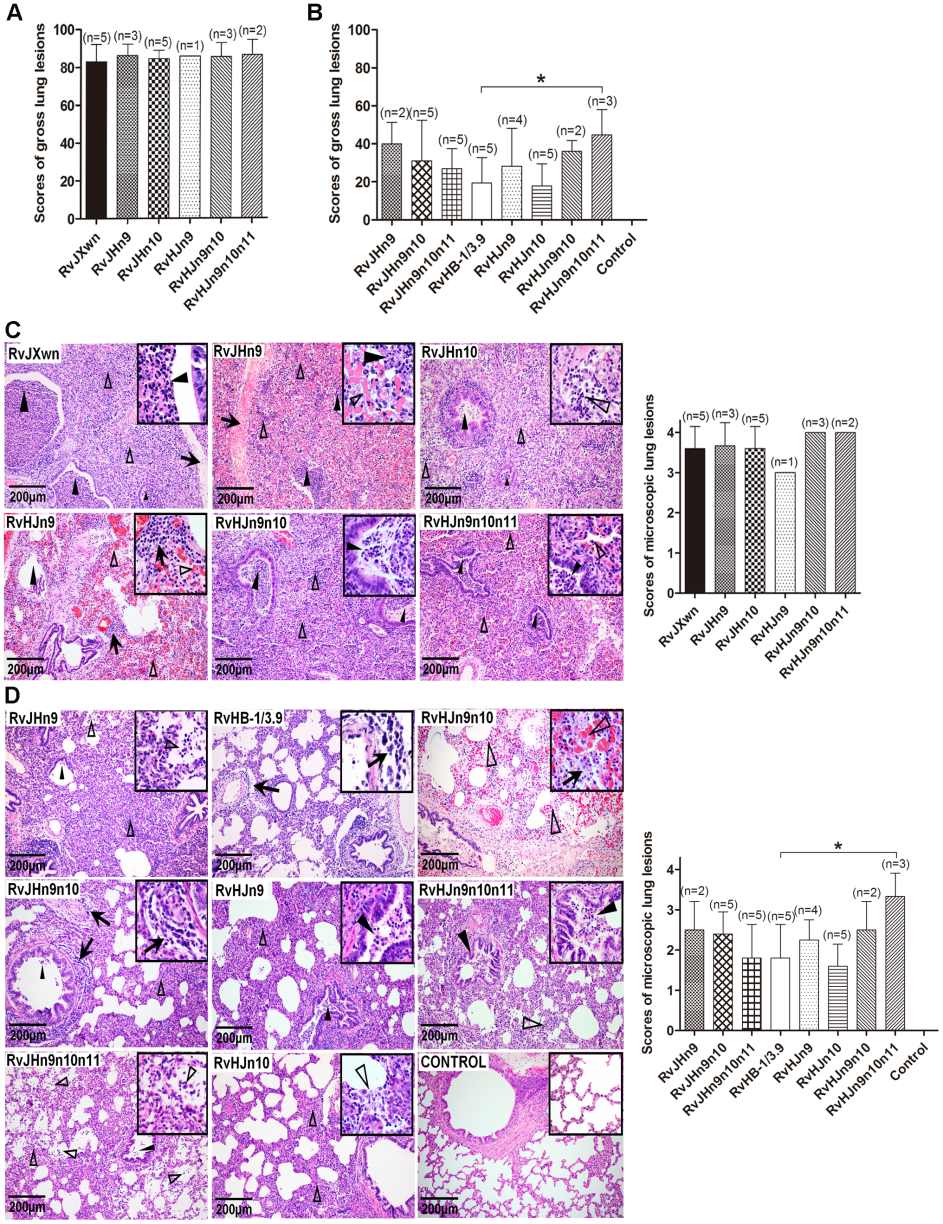
Scores of gross lung lesions and microscopic lung lesions of piglets inoculated with the rescued viruses. The mean scores of gross lung lesions, (A) from dead piglets during the experiment and (B) from euthanized piglets at the end of experiment in each group are shown. The gross lesions were graded based on the percentage of lung area affected. Lung sections were stained with hematoxylin and eosin (H&E). Representative pictures of the histopathological lung lesions and the average scores of dead piglets during the experiment (C) and euthanized piglets at the end of experiment (D) in each group. The microscopic lesions were scored based on the severity of interstitial pneumonia. Solid arrow indicates thickening of the interlobular septal or infiltration of inflammatory cells around the bronchiole. Solid triangle indicates inflammatory cells, necrotic debris and exfoliated epithelial cells infiltrate in the bronchiole. Triangle indicates hemorrhage or infiltration of inflammatory cells within alveolar septa, and alveolar spaces. Asterisk indicates significant differences in gross lesion scores and microscopic lesion scores between RvHB-1/3.9 and RvHJn9n10n11 (*P<0.05).

Necropsies and gross lung lesion examinations were immediately performed once the inoculated piglets died during the experiment. Similar to RvJXwn-infected piglets, all dead piglets in the chimeric virus-infected groups presented severe interstitial pneumonia with extensive and marked pulmonary edema, hemorrhage and consolidation (Supplemental Fig. S3A). Their mean scores of gross lung lesions showed no obvious differences (Fig. 8A). By the end of the experiment, the survived piglets were euthanized and necropsied. Moderate diffuse lung lesions were observed in RvJHn9-infected piglets, and scattered lung lesions were shown in the RvJHn9n10- and RvJHn9n10n11-infected piglets; in contrast, RvHJn9-, RvHJn9n10- or RvHJn9n10n11-infected piglets showed more severe interstitial pneumonia primarily at the cranial, middle lobes than RvHJn10- or RvHB-1/3.9-infected piglets (Supplemental Fig. S3B). Meanwhile, RvHJn9n10n11-infected piglets had a statistically higher mean scores of gross lung lesions than RvHB-1/3.9-infected piglets (*P*<0.05) (Fig. 8B).

**Figure 9:**
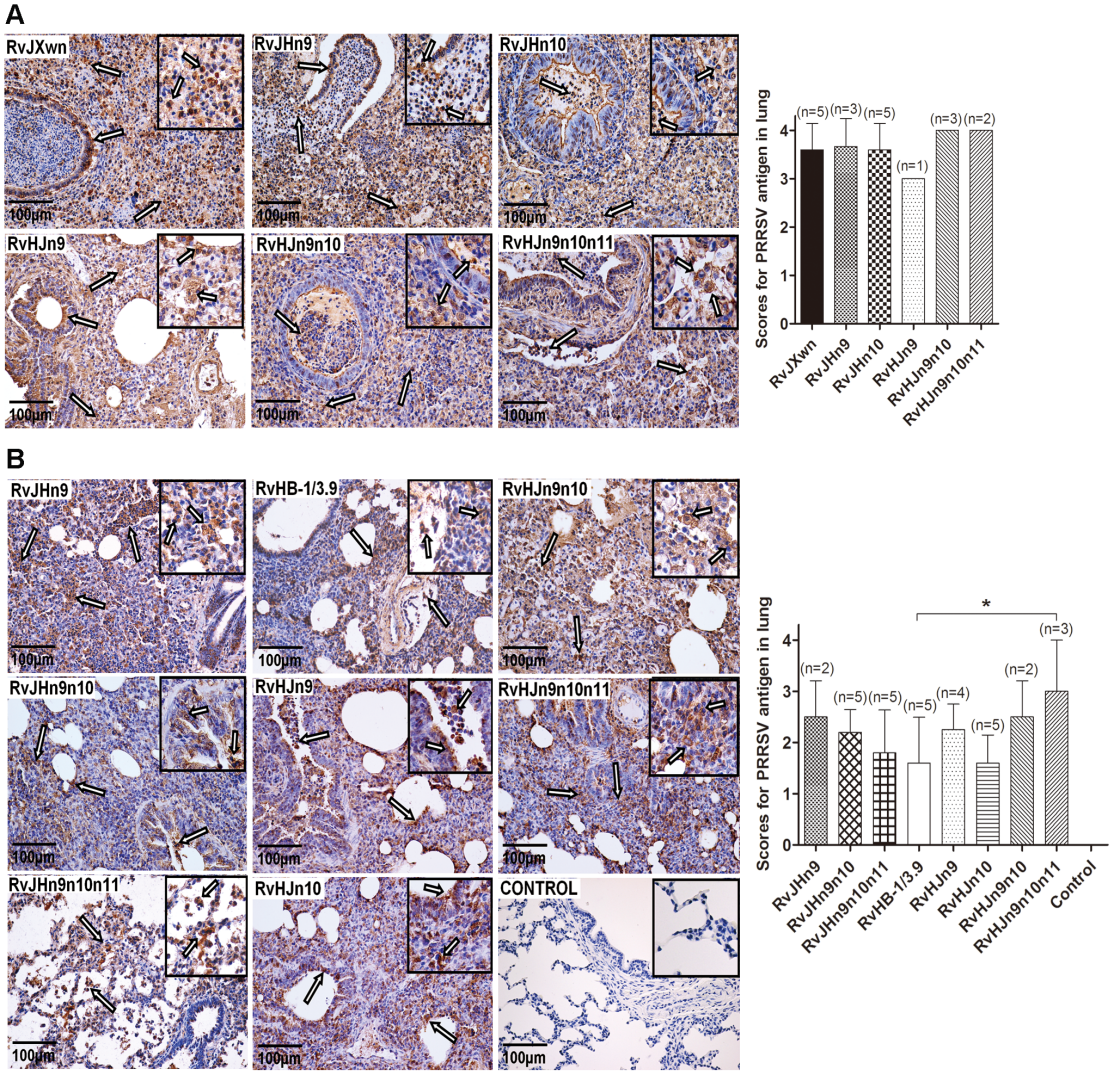
Immunohistochemical examination of inoculated piglets for PRRSV antigen. Lung sections were examined by immunohistochemistry (IHC) using monoclonal antibodies (SDOW17) specific for the N protein of PRRSV, and numbers of positive cells in lungs were scored. Representative pictures of immunohistochemistry examinations and mean scores of lungs of the dead piglets during the experiment (A) and of euthanized piglets by the end of experiment (B) in each group are shown. The macrophages stain intensely dark brown for the PRRSV antigen. Hollow arrow indicates positive signals in macrophages within or around alveolus and bronchus. Asterisk indicates a significant difference in IHC scores between RvHB-1/3.9 and RvHJn9n10n11 (*P<0.05).

The microscopic lesions of lungs were observed, and PRRSV antigens in lungs were examined by immunohistochemistry. Similar to RvJXwn, the lungs of the dead piglets in the chimeric virus-infected groups during the experimental period exhibited severe histopathological changes characterized by the complete disappearance of lung structure, thickening of the interlobular septal, a number of inflammatory cells and necrotic debris infiltration within both the alveolar spaces and bronchioleshared (Fig. 8C). Moreover, immunohistochemical staining showed that their lungs were full of PRRSV-positive signals, which were generally located in the alveolar and septa macrophages around bronchia, bronchiole, and alveolar septa (Fig. 9A). The average histopathological and immunohistochemical scores of those groups showed no obvious differences (Fig. 8C and 9A). In addition, the lungs of survived piglets in the RvJHn9-, RvJHn9n10- or RvJHn9n10n11-infected group displayed a gradually alleviated histopathological change; some slight microscopic lesions of lungs could be observed in RvHJn10- and RvHB-1/3.9-infected piglets. In contrast, the piglets exposed to RvHJn9, RvHJn9n10 or RvHJn9n10n11 showed increasingly severe histopathological changes (Fig. 8D) and PRRSV-positive signals closely correlated with the degree of histopathological lesions in lungs (Fig. 9B). The histopathological and immunohistochemical scores of the RvHJn9n10n11-infected group were both significantly higher than those of the RvHB-1/3.9-infected group (*P*<0.05) (Fig. 8D and 9B). No gross or microscopic lesions and PRRSV-positive signals were observed in the lungs of control piglets. These results indicated that RvJHn9n10 and RvJHn9n10n11 caused less severe histopathological lesions of lungs than RvJXwn, whereas RvHJn9n10 and RvHJn9n10n11 could remarkably lead to more severe histopathological lesions of lungs than RvHB-1/3.9.

Taken together, the above results indicated that the Nsp9- and Nsp10-coding regions of HP-PRRSV emerging in China contribute to its increased pathogenicity and fatal virulence for piglets.

### Nsp9 and Nsp10 together affect the virus growth *in vivo*

**Figure 10:**
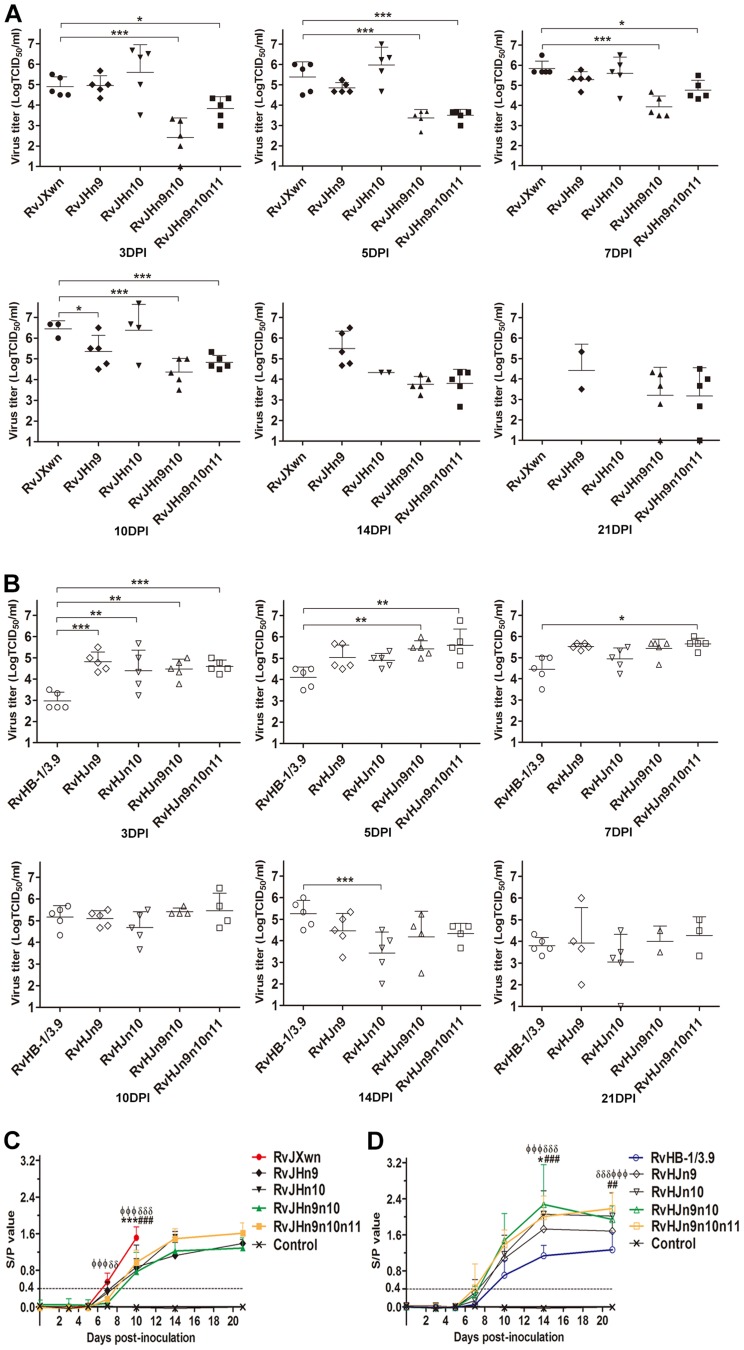
Viral loads and antibody kinetics in the sera of piglets inoculated with the rescued viruses. Virus titers in the sera of piglets inoculated with chimeric viruses with the RvJXwn backbone (A) and with the RvHB-1/3.9 backbone (B) are shown. Virus titers were determined by microtitration infectivity assay. The data are shown as the means ± standard deviations (error bars). Asterisk indicates significant differences in viral loads between chimeric virus and its parental backbone virus RvJXwn or RvHB-1/3.9 (*P<0.05; **P<0.01; ***P<0.001). The antibody kinetics of piglets inoculated with chimeric viruses with the RvJXwn backbone (C) and with the RvHB-1/3.9 backbone (D) are shown. The antibodies specific for PRRSV were detected using an IDEXX Herdchek PRRS 2XR ELISA kit, and the antibody level was expressed as a sample value/positive value (S/P) ratio. A ratio of ≥0.4 was regarded as seroconversion. Asterisk indicates significant differences in the antibody level between RvJXwn and RvJHn9 or between RvHB-1/3.9 and RvHJn9 (*P<0.05; ***P<0.001). Pound (#) indicates a significant difference between RvJXwn and RvJHn10 or between RvHB-1/3.9 and RvHJn10 (##*P*<0.01; ###*P*<0.001). Phi (Φ) indicates significant difference between RvJXwn and RvJHn9n10 or between RvHB-1/3.9 and RvHJn9n10 (ΦΦΦ*P*<0.001). Delta (δ) indicates significant difference between RvJXwn and RvJHn9n10n11 or between RvHB-1/3.9 and RvHJn9n10n11 (δδ*P*<0.01; δδδ*P*<0.001).

The virus loads in the sera of inoculated animals were examined using a microtitration assay. As shown in Fig. 10A, the virus titers in the sera of the RvJHn9-infected group were lower than those titers of the RvJXwn-infected group after day 3 pi, with significant differences on day 10 pi (*P*<0.05), whereas the viremia of RvJHn10-infected group was similar to those titers of the RvJXwn-infected group. Moreover the virus loads of the RvJHn9n10-infected piglets were significantly lower than those titers of the RvJXwn-infected group (*P*<0.001) and of the RvJHn9- and RvJHn10-infected groups at the majority of time points (*P*<0.05, *P*<0.01, *P*<0.001). Similarly, a remarkable reduction of virus loads in the RvJHn9n10n11-infected group was observed compared with the RvJXwn-infected group (*P*<0.001, *P*<0.05). As shown in Fig. 10B, RvHJn9 had a higher level of virus loads than RvHB-1/3.9 *in vivo* from day 3 to 7 pi, with significant differences on day 3 pi (*P*<0.001). Additionally, the virus load in the sera of RvHJn10-infected piglets was slightly higher than that of RvHB-1/3.9 on day 3 pi (*P*<0.01), but subsequently declined with a lower level on day 14 pi (*P*<0.001). In contrast, compared with RvHB-1/3.9, the virus loads of RvHJn9n10-infected piglets were obviously higher, with significant differences on days 3 and 5 pi (*P*<0.01); likewise, the RvHJn9n10n11-infected group showed higher virus titers with significant differences on days 3, 5, and 7 pi (*P*<0.05, *P*<0.001). The above data suggested that the exchanging of both Nsp9- and Nsp10-coding regions of RvJXwn by RvHB-1/3.9 could remarkably reduce the virus replication efficiency *in vivo*, and in contrast, the replacing of the corresponding regions of RvHB-1/3.9 by RvJXwn could increase the virus replication efficiency *in vivo* at a higher level.

The specific antibodies against the PRRSV N protein in the sera of infected pigs were measured using a commercial IDEXX ELISA kit. As shown in Fig. 10C, only one piglet in the RvJHn9- or RvJHn10-infected group became seroconverted, whereas the RvJXwn-infected group had four piglets seroconverted on day 7 pi. However, the piglets in the RvJHn9n10- or RvJHn9n10n11-infected groups were seropositive until day 10 pi, and meanwhile, their antibody levels were remarkably lower than those levels of the RvJXwn-infected group. In contrast, the RvHJn10- and RvHB-1/3.9-infected piglets were all seropositive on day 10 pi, and one piglet in the RvHJn9-, RvHJn9n10- or RvHJn9n10n11-infected groups was seroconverted as early as day 7 pi. Meanwhile, the antibody level of the RvHJn9n10-infected group was significantly higher than that of the RvHB-1/3.9-infected group on days 14 and 21 pi (*P*<0.001) (Fig. 10D). The control group remained seronegative during the entire experiment. These data further distinctly demonstrated that the Nsp9- and Nsp10-coding regions of HP-PRRSV together were closely related to the virus replication efficiency *in vivo*.

## Discussion

Highly pathogenic PRRSV has extensively prevailed in China and has resulted in great economic loss to the pig industry since 2006 [50]–[52]. HP-PRRSV still circulates in the field as the dominant virus, which may increase the diversity of PRRSV due to the possibility of viral recombination [63], [64]. Our previous studies have confirmed that the discontinued 30-amino-acid deletion in the Nsp2-coding region, which is the molecular marker of HP-PRRSV, is not directly related to its virulence [40]. It is necessary to explore which region(s) of the virus genome contribute to the pathogenicity and virulence of HP-PRRSV.

In the present study, large fragments of the genome were initially swapped between HP-PRRSV RvJXwn and LP-PRRSV RvHB-1/3.9 by reverse genetic operation to analyze the possible contributor of pathogenicity. Our data indicated that swapping the 5′UTR+ORF1a between RvJXwn and RvHB-1/3.9 could simultaneously impair the replication efficiency and pathogenicity of the chimeric viruses with either backbone. Meanwhile, the ORF1b showed obvious effects on the viral replication efficiency *in vitro* and *in vivo* and on the virulence for piglets, whereas the SP-coding region of HP-PRRSV did not contribute to its fatal virulence. To further explore the essential virulence determinants within the ORF1b region, we individually or simultaneously constructed and rescued twelve chimeric viruses with exchanged Nsp-coding regions. The pathogenicity for piglets and the *in vitro* and *in vivo* replication characteristics of the chimeric viruses were systematically investigated. The results showed that the replacement of Nsp9- and Nsp10-coding regions of RvJXwn together by RvHB-1/3.9 could greatly attenuate the virulence of RvJXwn. In contrast, the substitution of the corresponding regions of RvHB-1/3.9 by RvJXwn could remarkably increase the virulence of the chimeric virus. Therefore, our sufficient findings suggest that Nsp9- and Nsp10-coding region together contribute to the increased pathogenicity and fatal virulence of Chinese HP-PRRSV.

Two previous studies focused on exploring the virulence-associated genes or regions of PRRSV by using reverse genetic techniques [48], [49]. Kwon and his colleagues systematically substituted a series of small regions of the highly virulent PRRSV strain FL-12 by corresponding parts from the attenuated vaccine strain PrimePac. Using the reproductive failure model in sows, their findings indicated that the nonstructural proteins, particularly Nsp3–8, might play more important roles than structural proteins in the vaccine attenuation [49]. Our present findings showed that exchanging the 5′UTR+ORF1a (containing Nsp1 to Nsp8) could remarkably impair the fatal virulence of RvJXwn for piglets, but did not affect the pathogenicity of LP-PRRSV RvHB-1/3.9; however, exchanging the ORF1b region could not only enhance the virulence of LP-PRRSV but also reduce the virulence of HP-PRRSV. Although these two regions were both related to the reduction of virulence, we were more concerned regarding the region that contributed to the increased virulence. In our study, giving increased virulence is more important than decreasing virulence. Kwon and his colleagues also showed that the substitution of Nsp9 alone or Nsp10–12 together could impair the *in vitro* growth of the chimeric viruses, indicating that these nonstructural proteins may relate to the viral replication rate [49]. Our findings also showed that the replacement of Nsp9 of RvJXwn alone by RvHB-1/3.9 could obviously reduce the fatal virulence for piglets, although this replacement was less effective compared with the replacement of Nsp9 and Nsp10 together. In another similar study, the authors interchanged 5′UTR+ORF1ab or SP+3′UTR between highly pathogenic MN184 and vaccine strain PRRS MLV. The results of animal experiment demonstrated that both the region individually contributed to the attenuation of MN184 and that the 5-terminal fragment had greater effect on viral replication and lung lesion scores for pigs than the 3-terminal fragment [48]. Likewise, in our study, we found that ORF1a and ORF1b both had significant effects on the virulence attenuation of RvJXwn; however, the effect of the SP-coding region was not obvious, although the survival time of RvJHSP-infected piglets was prolonged, with 100% mortality of the infected piglets. When comparing the sequence of structural proteins among these viruses, HB-1/3.9 shares 98.13% nucleotide identity with JXwn06, which is significantly higher than the homology (90.0%) between MN184 and MLV. Thus, the structural protein might relate to the attenuation of MLV, but did not contribute to the increased fatal virulence of HP-PRRSV. This finding should be the major concern for the two different outcomes. In addition to analyzing the virulence-associated determining regions of PRRSV by reverse genetic operations, many studies were also conducted by comparing the genomic sequences between attenuated vaccine strains and their parental viruses [42], [46], [65], [66]. However, their results were different or even conflicted with each other due to strain-specific variation. Most of the above-mentioned previous studies analyzed the virulence of PRRSV by considering how these viruses were attenuated via serial passages in the permissive cells; thus, the possibility that the attenuation was caused by the incompatibility or imperfect matching of viral genes or UTR derived from the two different heterologous parental strains rather than caused by the true attenuation in a virulence determinant should be ruled out. This possibility can also be used to explain why both RvJH1a and RvHJ1a in our study were attenuated compared with their parental backbone viruses. In our study, we chose a different model to determine which region(s) within the genome contributes to the fatal virulence of HP-PRRSV by using two natural strains with distinct virulence. RvHB-1/3.9 as a LP-PRRSV shares 97.4% identity of the entire genome with HP-PRRSV RvJXwn; however, their virulence for piglets is completely different. Our previous studies have shown that RvJXwn is fatal for piglets, whereas RvHB-1/3.9 is not fatal [40]. Thus, using this model, we can distinguish the nonspecific effects due to foreign sequence insertion or to nucleotide mutation from the actual functional differences between virus strains; therefore, the virulence contribution can be solidly confirmed once any genes or regions from RvJXwn are able to increase the mortality of the infected piglets by the chimeric viruses with the RvHB-1/3.9 backbone.

As is well known, the pathogenicity of viruses is complicated and is dependent on multiple factors, such as viral propagation ability *in vivo*, tissue and/or cell tropism, immune escape and immune modification, as well as secondary bacterial infections. Many studies have indicated that the infection of highly virulent PRRSVs, such as Chinese HP-PRRSV and Lena, subtypes 3 strain of genotype 1, is usually accompanied by longer periods of viremia, increased severity of clinical signs, increased mortality, and significantly higher viral loads in blood and in tissues [40], [55], [67]–[69]. Additionally, different virulent PRRSVs may have different tissue and cell tropism. Our recent research found that HP-PRRSV JXwn06 infection exhibited more extensive tissue tropism for pigs than HB-1/3.9 [70]. A recent study showed that the highly pathogenic PRRSV Lena could infect more subtypes of macrophages than the early European strain LV did, which may result in more severe pathological changes [56]. Meanwhile, different virulent strains can induce different level of cytokines. Compared with the low virulence strain CH1a, Chinese HP-PRRSV HuN4 could induce a higher level of inflammatory cytokines, which might be closely associated with its marked damage on tissues and on organs [71]. Additionally, some studies showed that different genotypes (1 and 2) of PRRSV were able to induce different patterns of IL-10 and TNF-a, which, therefore, caused different outcomes of the infection [72]–[74]. Moreover, HP-PRRSV infection is usually accompanied by severe secondary bacterial infections, primarily including *Haemophilus parasuis* and *Streptococcus suis* infections [67]. We also observed that some dead piglets presented visible gross lesions, such as pericarditis and fibrinous pneumonia, which were caused by secondary bacterial infections during the experiment; this finding could be an important factor that exacerbates the illness and accelerates the death of the inoculated piglets. In this study, when Nsp9- and Nsp10-coding regions were exchanged together, RvJHn9n10 had significantly lower replication efficiency *in vitro* and *in vivo* and lower virulence for piglets than its parental virus, RvJXwn; In contrast, RvHJn9n10 displayed remarkably higher virus titers *in vitro* and *in vivo* and higher pathogenicity for piglets than its parental virus, RvHB-1/3.9. Nsp9, which contains RdRp, is regarded as a crucial motor for viral RNA replication [27], and Nsp10, which encodes RNA helicase, is another key enzyme that directly participates in RNA synthesis [28], [29]. Nsp9 and Nsp10 of EAV are assembled into a membrane-associated viral replication and transcription complex (RTC) [75], [76], which mediates both the genome replication and synthesis of a nested set of subgenomic (sg) mRNAs. Considering that both of these regions encode the essential key enzymes directly participating in genomic replication and transcription, not surprisingly, our results showing that Nsp9 and Nsp10 of Chinese HP-PRRSV were closely related to the increased replication efficiency are in agreement with their functions. Similar findings have been documented in some important human viruses. In influenza virus, the viral RNA polymerase complex has been proven to be related to the high pathogenicity and *in vivo* replication efficiency, which promotes optimal growth in the lower respiratory tract and respiratory droplet transmission in ferrets [77], [78]. Some researchers have found that the replication-associated proteins—NS3 (RNA helicase), NS4B or NS5 (RdRp) of dengue virus correlated with its *in vivo* virulence through affecting viral RNA synthesis, and consequently the degree of central nervous system damage [79]–[81]. Moreover, one single mutation in the NS3 of West Nile virus could remarkably increase its virulence, which is likely due to increased viral replication efficiency [82]. Meanwhile, the interaction between viral RdRp and viral RNA helicase is a universal phenomenon in many viruses, which is necessary for stimulating viral replication efficiency [83]–[86]. However, there is little knowledge regarding the crystal structure and function of PRRSV Nsp9 and Nsp10. Whether these proteins interact directly or indirectly through the help of other viral proteins or host cellular proteins should be further explored.

By aligning the amino acid sequences of the Nsp9 and Nsp10 regions between JXwn06 and HB-1/3.9 or among other Chinese HP-PRRSV strains, Vietnam HP-PRRSV, Laos HP-PRRSV, and Chinese LP-PRRSV strains, we found that there were 4 amino acids that were different in Nsp9 and 5 amino acids that were different in Nsp10 between JXwn06 and HB-1/3.9. Interestingly, one characteristic, a conserved amino acid difference in Nsp9 or Nsp10 between all HP-PRRSV and the Chinese LP-PRRSV strains was found, namely the amino acid at the position 544 in Nsp9 is alanine (A) in LP-PRRSV, whereas this amino acid is threonine (T) in HP-PRRSV, and the amino acid at the position 408 in Nsp10 is lysine (K) in LP-PRRSV, whereas this amino acid is arginine (R) in HP-PRRSV (Supplemental Fig. S4). Analyzing the possible roles of these two amino acids in the enhanced replication efficiency and virulence mediated by Nsp9 and Nsp10 will be of significance for HP-PRRSV pathogenesis. Based on the high identity and common conserved amino acid difference of Nsp9 and Nsp10 between those HP-PRRSV strains, Nsp9 and Nsp10 may likely be the fatal virulence determinants for other Asia HP-PRRSV, not only for Chinese ones. The European highly pathogenic PRRSV strain, Lena, has been recognized to exhibit enhanced replication efficiency and cause similar clinical conditions to Chinese HP-PRRSV [55]. Thus, investigating the role of Nsp9 and Nsp10 in the increased pathogenicity of Lena is necessary for elucidating the mechanisms associated with the virulence of Lena, although Lena and Chinese HP-PRRSV belong to distinct genotypes of PRRSV, with lower amino acid identity in their Nsp9 and Nsp10. Our present study provides a way to analyze the molecular mechanism of the increased virulence of Lena.

Our present study demonstrated that Nsp9 and Nsp10 together contribute to the fatal virulence of HP-PRRSV emerging in China. Additionally, our findings also provide direct evidence that the high replication efficiency is the critical factor for HP-PRRSV virulence. Moreover, by giving the high replication capacity to LP-PRRSV, its pathogenicity for piglets could be remarkably increased. However, more detailed studies concerning the molecular mechanism affecting PRRSV replication by Nsp9 and Nsp10 are required to better understand the pathogenesis of PRRSV. To the best of our knowledge, our findings are not only the first unambiguous illumination concerning the key virulence determinant of Chinese HP-PRRSV but also help to elucidate the pathogenesis of this virus and to develop new drugs and vaccines against PRRSV infection in the future.

In summary, our present study indicates that i) ORF1b affects the fatal virulence of HP-PRRSV for piglet; ii) Nsp9 and Nsp10 together are closely related to the replication efficiency of HP-PRRSV *in vitro*; iii) Nsp9 and Nsp10 together contribute to the fatal virulence of HP-PRRSV for piglets; iv) Nsp9 and Nsp10 together affect the HP-PRRSV growth *in vivo*.

## Supporting Information

Figure S1 **Examination of the chimeric viruses with the swapped three large coding regions.** MARC-145 cells infected with a third-passage culture of the rescued viruses were fixed at 48 h postinoculation and examined by IFA using monoclonal antibodies (SDOW17) against the N protein of PRRSV.
(TIF)

Figure S2 **Examination of the chimeric viruses with the exchanged Nsp-coding regions within the ORF1b.** MARC-145 cells infected with a third-passage culture of the rescued viruses were fixed at 48 h postinoculation and examined by IFA using monoclonal antibodies (SDOW17) against the N protein of PRRSV.
(TIF)

Figure S3 **Gross lung lesions of piglets inoculated with the rescued viruses.** Shown are the gross lesions of lungs from dead piglets during the experiment (A) and from euthanized piglets by the end of experiment (B) in each group.
(TIF)

Figure S4 **Alignment of amino acids in the Nsp9 and Nsp10 of PRRSV.** (A) Nsp9. (B) Nsp10. Dots indicate conserved residues. The amino acid differences were determined based on the amino acid sequence of JXwn06 Nsp9 or Nsp10.
(TIF)

Table S1 **Primers used for construction and detection of the chimeric viruses.** ^a^ F denotes a forward PCR primer; R denotes reverse transcription or a reverse PCR primer. ^b^ Numbers refer to nucleotide positions within the genome of JXwn06 (GenBank accession no: EF641008) or HB-1/3.9 (GenBank accession no: EU360130), as indicated. ^c^ Restriction sites introduced by PCR are shown in boldface and specified in parentheses at the end of the sequence.
(DOC)

Table S2 **Clinical sign scoring system used for analyzing the pathogenicity of rescued viruses.** Usual condition: total score = GCS+RCS+NSS. If piglet died: total score = GCS+RCS+NSS+5. 0≤total score≤20.
(DOCX)

## References

[1] Albina E (1997) Epidemiology of porcine reproductive and respiratory syndrome (PRRS): an overview. Vet Microbiol 55: 309–316. 9220627 10.1016/s0378-1135(96)01322-3

[2] Pejsak Z, Stadejek T, Markowska-Daniel I (1997) Clinical signs and economic losses caused by porcine reproductive and respiratory syndrome virus in a large breeding farm. Vet Microbiol 55: 317–322. 9220628 10.1016/s0378-1135(96)01326-0

[3] Keffaber KK (1989) Reproductive failure of unknown etiology. Am Assoc Swine Pract Newsl 1.2: 9.

[4] Bilodeau R, Dea S, Sauvageau RA, Martineau GP (1991) ‘Porcine reproductive and respiratory syndrome’ in Quebec. Vet Rec 129: 102–103. 10.1136/vr.129.5.102 1926720

[5] Albina E, Baron T, Leforban Y (1992) Blue-eared pig disease in Brittany. Vet Rec 130: 58–59. 10.1136/vr.130.3.58 1546437

[6] Wensvoort G, Terpstra C, Pol JM, ter Laak EA, Bloemraad M, et al (1991) Mystery swine disease in The Netherlands: the isolation of Lelystad virus. Vet Q 13: 121–130. 1835211 10.1080/01652176.1991.9694296

[7] Collins JE, Benfield DA, Christianson WT, Harris L, Hennings JC, et al (1992) Isolation of swine infertility and respiratory syndrome virus (isolate ATCC VR-2332) in North America and experimental reproduction of the disease in gnotobiotic pigs. J Vet Diagn Invest 4: 117–126. 1616975 10.1177/104063879200400201

[8] Hopper SA, White ME, Twiddy N (1992) An outbreak of blue-eared pig disease (porcine reproductive and respiratory syndrome) in four pig herds in Great Britain. Vet Rec 131: 140–144. 1413421 10.1136/vr.131.7.140

[9] Botner A, Nielsen J, Bille-Hansen V (1994) Isolation of porcine reproductive and respiratory syndrome (PRRS) virus in a Danish swine herd and experimental infection of pregnant gilts with the virus. Vet Microbiol 40: 351–360. 7941298 10.1016/0378-1135(94)90122-8

[10] Kuwahara H, Nunoya T, Tajima M, Kato A, Samejima T (1994) An outbreak of porcine reproductive and respiratory syndrome in Japan. J Vet Med Sci 56: 901–909. 7865592 10.1292/jvms.56.901

[11] Garner MG, Whan IF, Gard GP, Phillips D (2001) The expected economic impact of selected exotic diseases on the pig industry of Australia. Rev Sci Tech 20: 671–685. 11732410 10.20506/rst.20.3.1303

[12] Neumann EJ, Kliebenstein JB, Johnson CD, Mabry JW, Bush EJ, et al (2005) Assessment of the economic impact of porcine reproductive and respiratory syndrome on swine production in the United States. J Am Vet Med Assoc 227: 385–392. 16121604 10.2460/javma.2005.227.385

[13] Meulenberg JJ, Hulst MM, de Meijer EJ, Moonen PL, den Besten A, et al (1993) Lelystad virus, the causative agent of porcine epidemic abortion and respiratory syndrome (PEARS), is related to LDV and EAV. Virology 192: 62–72. 8517032 10.1006/viro.1993.1008 PMC7173055

[14] Mardassi H, Mounir S, Dea S (1994) Identification of major differences in the nucleocapsid protein genes of a Quebec strain and European strains of porcine reproductive and respiratory syndrome virus. J Gen Virol 75: 681–685. 8126467 10.1099/0022-1317-75-3-681

[15] Stadejek T, Oleksiewicz MB, Scherbakov AV, Timina AM, Krabbe JS, et al (2008) Definition of subtypes in the European genotype of porcine reproductive and respiratory syndrome virus: nucleocapsid characteristics and geographical distribution in Europe. Arch Virol 153: 1479–1488. 18592131 10.1007/s00705-008-0146-2

[16] Shi M, Lam TT, Hon CC, Murtaugh MP, Davies PR, et al (2010) Phylogeny-based evolutionary, demographical, and geographical dissection of North American type 2 porcine reproductive and respiratory syndrome viruses. J Virol 84: 8700–8711. 20554771 10.1128/JVI.02551-09 PMC2919017

[17] Conzelmann KK, Visser N, Van Woensel P, Thiel HJ (1993) Molecular characterization of porcine reproductive and respiratory syndrome virus, a member of the arterivirus group. Virology 193: 329–339. 8438574 10.1006/viro.1993.1129 PMC7131490

[18] Snijder EJ, Meulenberg JJ (1998) The molecular biology of arteriviruses. J Gen Virol 79: 961–979. 9603311 10.1099/0022-1317-79-5-961

[19] Firth AE, Zevenhoven-Dobbe JC, Wills NM, Go YY, Balasuriya UB, et al (2011) Discovery of a small arterivirus gene that overlaps the GP5 coding sequence and is important for virus production. J Gen Virol 92: 1097–1106. 21307223 10.1099/vir.0.029264-0 PMC3139419

[20] Johnson CR, Griggs TF, Gnanandarajah J, Murtaugh MP (2011) Novel structural protein in porcine reproductive and respiratory syndrome virus encoded by an alternative ORF5 present in all arteriviruses. J Gen Virol 92: 1107–1116. 21307222 10.1099/vir.0.030213-0 PMC3139420

[21] Snijder EJ, Wassenaar AL, Spaan WJ (1994) Proteolytic processing of the replicase ORF1a protein of equine arteritis virus. J Virol 68: 5755–5764. 8057457 10.1128/jvi.68.9.5755-5764.1994 PMC236979

[22] den Boon JA, Faaberg KS, Meulenberg JJ, Wassenaar AL, Plagemann PG, et al (1995) Processing and evolution of the N-terminal region of the arterivirus replicase ORF1a protein: identification of two papainlike cysteine proteases. J Virol 69: 4500–4505. 7769711 10.1128/jvi.69.7.4500-4505.1995 PMC189193

[23] van Dinten LC, Wassenaar AL, Gorbalenya AE, Spaan WJ, Snijder EJ (1996) Processing of the equine arteritis virus replicase ORF1b protein: identification of cleavage products containing the putative viral polymerase and helicase domains. J Virol 70: 6625–6633. 8794297 10.1128/jvi.70.10.6625-6633.1996 PMC190703

[24] Wassenaar AL, Spaan WJ, Gorbalenya AE, Snijder EJ (1997) Alternative proteolytic processing of the arterivirus replicase ORF1a polyprotein: evidence that NSP2 acts as a cofactor for the NSP4 serine protease. J Virol 71: 9313–9322. 9371590 10.1128/jvi.71.12.9313-9322.1997 PMC230234

[25] Fang Y, Snijder EJ (2010) The PRRSV replicase: exploring the multifunctionality of an intriguing set of nonstructural proteins. Virus Res 154: 61–76. 20696193 10.1016/j.virusres.2010.07.030 PMC7114499

[26] Fang Y, Treffers EE, Li Y, Tas A, Sun Z, et al (2012) Efficient -2 frameshifting by mammalian ribosomes to synthesize an additional arterivirus protein. Proc Natl Acad Sci U S A 109: E2920–2928. 23043113 10.1073/pnas.1211145109 PMC3491471

[27] Beerens N, Selisko B, Ricagno S, Imbert I, van der Zanden L, et al (2007) De novo initiation of RNA synthesis by the arterivirus RNA-dependent RNA polymerase. J Virol 81: 8384–8395. 17537850 10.1128/JVI.00564-07 PMC1951334

[28] van Dinten LC, van Tol H, Gorbalenya AE, Snijder EJ (2000) The predicted metal-binding region of the arterivirus helicase protein is involved in subgenomic mRNA synthesis, genome replication, and virion biogenesis. J Virol 74: 5213–5223. 10799597 10.1128/jvi.74.11.5213-5223.2000 PMC110875

[29] Bautista EM, Faaberg KS, Mickelson D, McGruder ED (2002) Functional properties of the predicted helicase of porcine reproductive and respiratory syndrome virus. Virology 298: 258–270. 12127789 10.1006/viro.2002.1495 PMC7130902

[30] Nedialkova DD, Ulferts R, van den Born E, Lauber C, Gorbalenya AE, et al (2009) Biochemical characterization of arterivirus nonstructural protein 11 reveals the nidovirus-wide conservation of a replicative endoribonuclease. J Virol 83: 5671–5682. 19297500 10.1128/JVI.00261-09 PMC2681944

[31] Beura LK, Sarkar SN, Kwon B, Subramaniam S, Jones C, et al (2010) Porcine reproductive and respiratory syndrome virus nonstructural protein 1beta modulates host innate immune response by antagonizing IRF3 activation. J Virol 84: 1574–1584. 19923190 10.1128/JVI.01326-09 PMC2812326

[32] Shi X, Wang L, Li X, Zhang G, Guo J, et al (2011) Endoribonuclease activities of porcine reproductive and respiratory syndrome virus nsp11 was essential for nsp11 to inhibit IFN-beta induction. Mol Immunol 48: 1568–1572. 21481939 10.1016/j.molimm.2011.03.004 PMC7112683

[33] Sun Y, Han M, Kim C, Calvert JG, Yoo D (2012) Interplay between interferon-mediated innate immunity and porcine reproductive and respiratory syndrome virus. Viruses 4: 424–446. 22590680 10.3390/v4040424 PMC3347317

[34] Bautista EM, Meulenberg JJ, Choi CS, Molitor TW (1996) Structural polypeptides of the American (VR-2332) strain of porcine reproductive and respiratory syndrome virus. Arch Virol 141: 1357–1365. 8774694 10.1007/BF01718837

[35] Van Breedam W, Delputte PL, Van Gorp H, Misinzo G, Vanderheijden N, et al (2010) Porcine reproductive and respiratory syndrome virus entry into the porcine macrophage. J Gen Virol 91: 1659–1667. 20410315 10.1099/vir.0.020503-0

[36] Vanhee M, Costers S, Van Breedam W, Geldhof MF, Van Doorsselaere J, et al (2010) A variable region in GP4 of European-type porcine reproductive and respiratory syndrome virus induces neutralizing antibodies against homologous but not heterologous virus strains. Viral Immunol 23: 403–413. 20712485 10.1089/vim.2010.0025 PMC2928701

[37] Van Breedam W, Van Gorp H, Zhang JQ, Crocker PR, Delputte PL, et al (2010) The M/GP(5) glycoprotein complex of porcine reproductive and respiratory syndrome virus binds the sialoadhesin receptor in a sialic acid-dependent manner. PLoS Pathog 6: e1000730. 20084110 10.1371/journal.ppat.1000730 PMC2799551

[38] Halbur PG, Paul PS, Frey ML, Landgraf J, Eernisse K, et al (1995) Comparison of the pathogenicity of two US porcine reproductive and respiratory syndrome virus isolates with that of the Lelystad virus. Vet Pathol 32: 648–660. 8592800 10.1177/030098589503200606

[39] Mengeling WL, Vorwald AC, Lager KM, Brockmeier SL (1996) Comparison among strains of porcine reproductive and respiratory syndrome virus for their ability to cause reproductive failure. Am J Vet Res 57: 834–839. 8725809

[40] Zhou L, Zhang J, Zeng J, Yin S, Li Y, et al (2009) The 30-amino-acid deletion in the Nsp2 of highly pathogenic porcine reproductive and respiratory syndrome virus emerging in China is not related to its virulence. J Virol 83: 5156–5167. 19244318 10.1128/JVI.02678-08 PMC2682102

[41] Lunney JK, Benfield DA, Rowland RR (2010) Porcine reproductive and respiratory syndrome virus: an update on an emerging and re-emerging viral disease of swine. Virus Res 154: 1–6. 20951175 10.1016/j.virusres.2010.10.009 PMC7172856

[42] Yang SX, Kwang J, Laegreid W (1998) Comparative sequence analysis of open reading frames 2 to 7 of the modified live vaccine virus and other North American isolates of the porcine reproductive and respiratory syndrome virus. Arch Virol 143: 601–612. 9572560 10.1007/s007050050316

[43] Oleksiewicz MB, Botner A, Nielsen J, Storgaard T (1999) Determination of 5′-leader sequences from radically disparate strains of porcine reproductive and respiratory syndrome virus reveals the presence of highly conserved sequence motifs. Arch Virol 144: 981–987. 10416379 10.1007/s007050050560 PMC7086836

[44] Storgaard T, Oleksiewicz M, Botner A (1999) Examination of the selective pressures on a live PRRS vaccine virus. Arch Virol 144: 2389–2401. 10664392 10.1007/s007050050652

[45] Allende R, Kutish GF, Laegreid W, Lu Z, Lewis TL, et al (2000) Mutations in the genome of porcine reproductive and respiratory syndrome virus responsible for the attenuation phenotype. Arch Virol 145: 1149–1161. 10948988 10.1007/s007050070115 PMC7086797

[46] Yuan S, Mickelson D, Murtaugh MP, Faaberg KS (2001) Complete genome comparison of porcine reproductive and respiratory syndrome virus parental and attenuated strains. Virus Res 74: 99–110. 11226578 10.1016/S0168-1702(00)00250-1 PMC7125765

[47] Grebennikova TV, Clouser DF, Vorwald AC, Musienko MI, Mengeling WL, et al (2004) Genomic characterization of virulent, attenuated, and revertant passages of a North American porcine reproductive and respiratory syndrome virus strain. Virology 321: 383–390. 15051397 10.1016/j.virol.2004.01.001

[48] Wang Y, Liang Y, Han J, Burkhart KM, Vaughn EM, et al (2008) Attenuation of porcine reproductive and respiratory syndrome virus strain MN184 using chimeric construction with vaccine sequence. Virology 371: 418–429. 17976680 10.1016/j.virol.2007.09.032

[49] Kwon B, Ansari IH, Pattnaik AK, Osorio FA (2008) Identification of virulence determinants of porcine reproductive and respiratory syndrome virus through construction of chimeric clones. Virology 380: 371–378. 18768197 10.1016/j.virol.2008.07.030

[50] Tian K, Yu X, Zhao T, Feng Y, Cao Z, et al (2007) Emergence of fatal PRRSV variants: unparalleled outbreaks of atypical PRRS in China and molecular dissection of the unique hallmark. PLoS One 2: e526. 17565379 10.1371/journal.pone.0000526 PMC1885284

[51] Zhou L, Yang H (2010) Porcine reproductive and respiratory syndrome in China. Virus Res 154: 31–37. 20659506 10.1016/j.virusres.2010.07.016

[52] Zhou L, Chen S, Zhang J, Zeng J, Guo X, et al (2009) Molecular variation analysis of porcine reproductive and respiratory syndrome virus in China. Virus Res 145: 97–105. 19559739 10.1016/j.virusres.2009.06.014

[53] Li Y, Wang X, Bo K, Wang X, Tang B, et al (2007) Emergence of a highly pathogenic porcine reproductive and respiratory syndrome virus in the Mid-Eastern region of China. Vet J 174: 577–584. 17869553 10.1016/j.tvjl.2007.07.032

[54] Zhou YJ, Hao XF, Tian ZJ, Tong GZ, Yoo D, et al (2008) Highly virulent porcine reproductive and respiratory syndrome virus emerged in China. Transbound Emerg Dis 55: 152–164. 18405338 10.1111/j.1865-1682.2008.01020.x

[55] Karniychuk UU, Geldhof M, Vanhee M, Van Doorsselaere J, Saveleva TA, et al (2010) Pathogenesis and antigenic characterization of a new East European subtype 3 porcine reproductive and respiratory syndrome virus isolate. BMC Vet Res 6: 30–39. 20525333 10.1186/1746-6148-6-30 PMC2898778

[56] Frydas IS, Verbeeck M, Cao J, Nauwynck HJ (2013) Replication characteristics of porcine reproductive and respiratory syndrome virus (PRRSV) European subtype 1 (Lelystad) and subtype 3 (Lena) strains in nasal mucosa and cells of the monocytic lineage: indications for the use of new receptors of PRRSV (Lena). Vet Res 44: 73–86. 24007551 10.1186/1297-9716-44-73 PMC3849772

[57] Zhang H, Guo X, Ge X, Chen Y, Sun Q, et al (2009) Changes in the cellular proteins of pulmonary alveolar macrophage infected with porcine reproductive and respiratory syndrome virus by proteomics analysis. J Proteome Res 8: 3091–3097. 19341299 10.1021/pr900002f

[58] Gao ZQ, Guo X, Yang HC (2004) Genomic characterization of two Chinese isolates of porcine respiratory and reproductive syndrome virus. Arch Virol 149: 1341–1351. 15221535 10.1007/s00705-004-0292-0

[59] Meng XJ, Paul PS, Halbur PG, Lum MA (1996) Characterization of a high-virulence US isolate of porcine reproductive and respiratory syndrome virus in a continuous cell line, ATCC CRL11171. J Vet Diagn Invest 8: 374–381. 8844584 10.1177/104063879600800317

[60] Fang Y, Rowland RR, Roof M, Lunney JK, Christopher-Hennings J, et al (2006) A full-length cDNA infectious clone of North American type 1 porcine reproductive and respiratory syndrome virus: expression of green fluorescent protein in the Nsp2 region. J Virol 80: 11447–11455. 16971421 10.1128/JVI.01032-06 PMC1642622

[61] Halbur PG, Paul PS, Frey ML, Landgraf J, Eernisse K, et al (1995) Comparison of the pathogenicity of two US porcine reproductive and respiratory syndrome virus isolates with that of the Lelystad virus. Vet Pathol 32: 648–660. 8592800 10.1177/030098589503200606

[62] Halbur PG, Paul PS, Frey ML, Landgraf J, Eernisse K, et al (1996) Comparison of the antigen distribution of two US porcine reproductive and respiratory syndrome virus isolates with that of the Lelystad virus. Vet Pathol 33: 159–170. 8801709 10.1177/030098589603300205

[63] Liu D, Zhou R, Zhang J, Zhou L, Jiang Q, et al (2011) Recombination analyses between two strains of porcine reproductive and respiratory syndrome virus in vivo. Virus Res 155: 473–486. 21167230 10.1016/j.virusres.2010.12.003

[64] Shi M, Holmes EC, Brar MS, Leung FC (2013) Recombination is associated with an outbreak of novel highly pathogenic porcine reproductive and respiratory syndrome viruses in China. J Virol 87: 10904–10907. 23885071 10.1128/JVI.01270-13 PMC3807407

[65] Opriessnig T, Halbur PG, Yoon KJ, Pogranichniy RM, Harmon KM, et al (2002) Comparison of molecular and biological characteristics of a modified live porcine reproductive and respiratory syndrome virus (PRRSV) vaccine (ingelvac PRRS MLV), the parent strain of the vaccine (ATCC VR2332), ATCC VR2385, and two recent field isolates of PRRSV. J Virol 76: 11837–11844. 12414926 10.1128/JVI.76.23.11837-11844.2002 PMC136866

[66] An TQ, Tian ZJ, Zhou YJ, Xiao Y, Peng JM, et al (2011) Comparative genomic analysis of five pairs of virulent parental/attenuated vaccine strains of PRRSV. Vet Microbiol 149: 104–112. 21111544 10.1016/j.vetmic.2010.11.001

[67] Guo B, Lager KM, Henningson JN, Miller LC, Schlink SN, et al (2013) Experimental infection of United States swine with a Chinese highly pathogenic strain of porcine reproductive and respiratory syndrome virus. Virology 435: 372–384. 23079105 10.1016/j.virol.2012.09.013 PMC7111980

[68] Johnson W, Roof M, Vaughn E, Christopher-Hennings J, Johnson CR, et al (2004) Pathogenic and humoral immune responses to porcine reproductive and respiratory syndrome virus (PRRSV) are related to viral load in acute infection. Vet Immunol Immunopathol 102: 233–247. 15507308 10.1016/j.vetimm.2004.09.010

[69] Petry DB, Lunney J, Boyd P, Kuhar D, Blankenship E, et al (2007) Differential immunity in pigs with high and low responses to porcine reproductive and respiratory syndrome virus infection. J Anim Sci 85: 2075–2092. 17468430 10.2527/jas.2006-721

[70] Li L, Zhao Q, Ge X, Teng K, Kuang Y, et al (2012) Chinese highly pathogenic porcine reproductive and respiratory syndrome virus exhibits more extensive tissue tropism for pigs. Virol J 9: 203–208. 22978380 10.1186/1743-422X-9-203 PMC3487961

[71] Han Z, Liu Y, Wang G, He Y, Hu S, et al (2013) Comparative analysis of immune responses in pigs to high and low pathogenic porcine reproductive and respiratory syndrome viruses isolated in China. Transbound Emerg Dis 6: 12190–12199. 10.1111/tbed.12190 24308664

[72] Lee SM, Schommer SK, Kleiboeker SB (2004) Porcine reproductive and respiratory syndrome virus field isolates differ in in vitro interferon phenotypes. Vet Immunol Immunopathol 102: 217–231. 15507307 10.1016/j.vetimm.2004.09.009 PMC7112598

[73] Gimeno M, Darwich L, Diaz I, de la Torre E, Pujols J, et al (2011) Cytokine profiles and phenotype regulation of antigen presenting cells by genotype-I porcine reproductive and respiratory syndrome virus isolates. Vet Res 42: 9–18. 21314968 10.1186/1297-9716-42-9 PMC3037899

[74] Baumann A, Mateu E, Murtaugh MP, Summerfield A (2013) Impact of genotype 1 and 2 of porcine reproductive and respiratory syndrome viruses on interferon-alpha responses by plasmacytoid dendritic cells. Vet Res 44: 33–42. 23675981 10.1186/1297-9716-44-33 PMC3672080

[75] Pedersen KW, van der Meer Y, Roos N, Snijder EJ (1999) Open reading frame 1a-encoded subunits of the arterivirus replicase induce endoplasmic reticulum-derived double-membrane vesicles which carry the viral replication complex. J Virol 73: 2016–2026. 9971782 10.1128/jvi.73.3.2016-2026.1999 PMC104444

[76] van Hemert MJ, de Wilde AH, Gorbalenya AE, Snijder EJ (2008) The in vitro RNA synthesizing activity of the isolated arterivirus replication/transcription complex is dependent on a host factor. J Biol Chem 283: 16525–16536. 18411274 10.1074/jbc.M708136200

[77] Watanabe T, Watanabe S, Shinya K, Kim JH, Hatta M, et al (2009) Viral RNA polymerase complex promotes optimal growth of 1918 virus in the lower respiratory tract of ferrets. Proc Natl Acad Sci U S A 106: 588–592. 19114663 10.1073/pnas.0806959106 PMC2626747

[78] Pascua PN, Song MS, Kwon HI, Lim GJ, Kim EH, et al (2013) The homologous tripartite viral RNA polymerase of A/swine/Korea/CT1204/2009(H1N2) influenza virus synergistically drives efficient replication and promotes respiratory-droplet transmission in ferrets. J Virol 87: 10552–10562. 23864624 10.1128/JVI.01333-13 PMC3807396

[79] Engel AR, Rumyantsev AA, Maximova OA, Speicher JM, Heiss B (2010) The neurovirulence and neuroinvasiveness of chimeric tick-borne encephalitis/dengue virus can be attenuated by introducing defined mutations into the envelope and NS5 protein genes and the 3′ non-coding region of the genome. Virology 405: 243–252. 20594569 10.1016/j.virol.2010.06.014 PMC2914112

[80] Grant D, Tan GK, Qing M, Ng JK, Yip A, et al (2011) A single amino acid in nonstructural protein NS4B confers virulence to dengue virus in AG129 mice through enhancement of viral RNA synthesis. J Virol 85: 7775–7787. 21632767 10.1128/JVI.00665-11 PMC3147917

[81] de Borba L, Strottmann DM, de Noronha L, Mason PW, Dos Santos CN (2012) Synergistic interactions between the NS3(hel) and E proteins contribute to the virulence of dengue virus type 1. PLoS Negl Trop Dis 6: e1624. 22530074 10.1371/journal.pntd.0001624 PMC3328427

[82] Brault AC, Huang CY, Langevin SA, Kinney RM, Bowen RA, et al (2007) A single positively selected West Nile viral mutation confers increased virogenesis in American crows. Nat Genet 39: 1162–1166. 17694056 10.1038/ng2097 PMC2291521

[83] Wang P, Wang Y, Zhao Y, Zhu Z, Yu J, et al (2010) Classical swine fever virus NS3 enhances RNA-dependent RNA polymerase activity by binding to NS5B. Virus Res 148: 17–23. 19951725 10.1016/j.virusres.2009.11.015

[84] Zhang C, Cai Z, Kim YC, Kumar R, Yuan F, et al (2005) Stimulation of hepatitis C virus (HCV) nonstructural protein 3 (NS3) helicase activity by the NS3 protease domain and by HCV RNA-dependent RNA polymerase. J Virol 79: 8687–8697. 15994762 10.1128/JVI.79.14.8687-8697.2005 PMC1168731

[85] Kapoor M, Zhang L, Ramachandra M, Kusukawa J, Ebner KE, et al (1995) Association between NS3 and NS5 proteins of dengue virus type 2 in the putative RNA replicase is linked to differential phosphorylation of NS5. J Biol Chem 270: 19100–19106. 7642575 10.1074/jbc.270.32.19100

[86] Johansson M, Brooks AJ, Jans DA, Vasudevan SG (2001) A small region of the dengue virus-encoded RNA-dependent RNA polymerase, NS5, confers interaction with both the nuclear transport receptor importin-beta and the viral helicase, NS3. J Gen Virol 82: 735–745. 11257177 10.1099/0022-1317-82-4-735

