# Correction: Nsp9 and Nsp10 Contribute to the Fatal Virulence of Highly Pathogenic Porcine Reproductive and Respiratory Syndrome Virus Emerging in China

**DOI:** 10.1371/journal.ppat.1004344

**Published:** 2014-08-08

**Authors:** The PLOS Pathogens Staff

The incorrect version of Figure 5 was published. The icons showing RvHJn9 and RvHJn11 in panel C are now consistent with the icons in panel G. Please see the corrected figure here.

**Figure 5:**
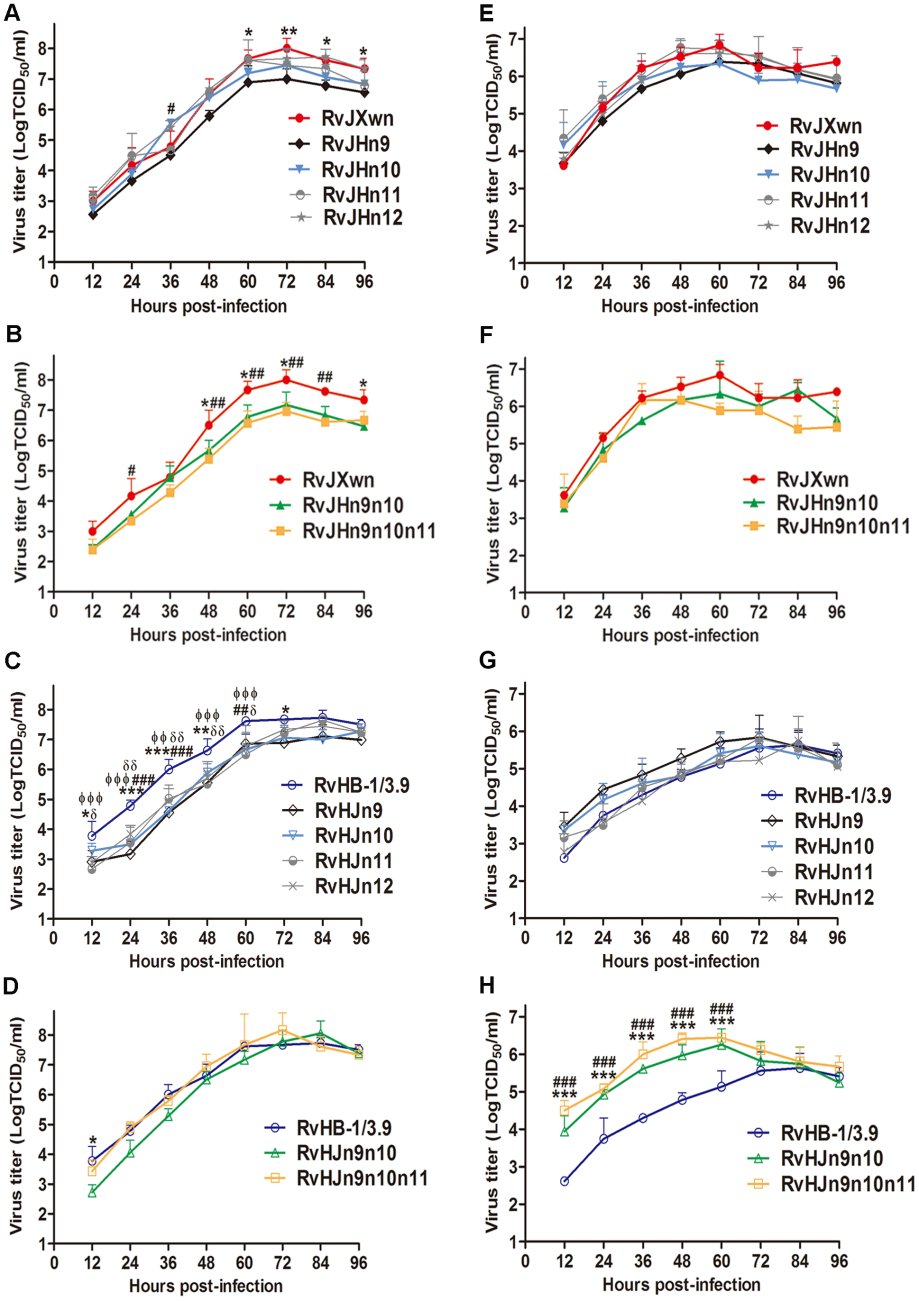
Growth kinetics of the rescued viruses with the exchanged Nsp-coding region within ORF1b. The growth curves of each chimeric virus, RvJXwn and RvHB-1/3.9 in MARC-145 cells (A, B, C and D) and in primary PAMs (E, F, G and H) are shown. Virus titers from 12 h to 96 h pi were determined by microtitration infectivity assays. The data are shown as the means ± standard deviations (error bars) from three independent trials. Asterisk (*) indicates a significant difference in virus titers between RvJXwn (red) and RvJHn9 (black), or RvJHn9n10 (green), or between RvHB-1/3.9 (blue) and RvHJn9 (black) or RvHJn9n10 (green) (*P<0.05; **P<0.01; ***P<0.001). Pound (#) indicates a significant difference between RvJXwn and RvJHn10 (light blue) or RvJHn9n10n11 (yellow), or between RvHB-1/3.9 and RvHJn10 (light blue) or RvHJn9n10n11 (yellow) (##*P*<0.01; ###*P*<0.001). Phi (Φ) indicates a significant difference between RvHB-1/3.9 and RvHJn11 (gray) (ΦΦ*P*<0.01; ΦΦΦ*P*<0.001). Delta (δ) indicates a significant difference between RvHB-1/3.9 and RvHJn12 (gray) (δ*P*<0.05; δδ*P*<0.01). doi:10.1371/journal.ppat.1004216.g005

The incorrect version of Figure 6 was published. The authors have changed the vertical arrangement of panel B, C, D, E and F to a horizontal style, and moved the graphs of clinical sign scores to the front of the graphs of average daily gains. Please see the corrected figure here.

**Figure 6:**
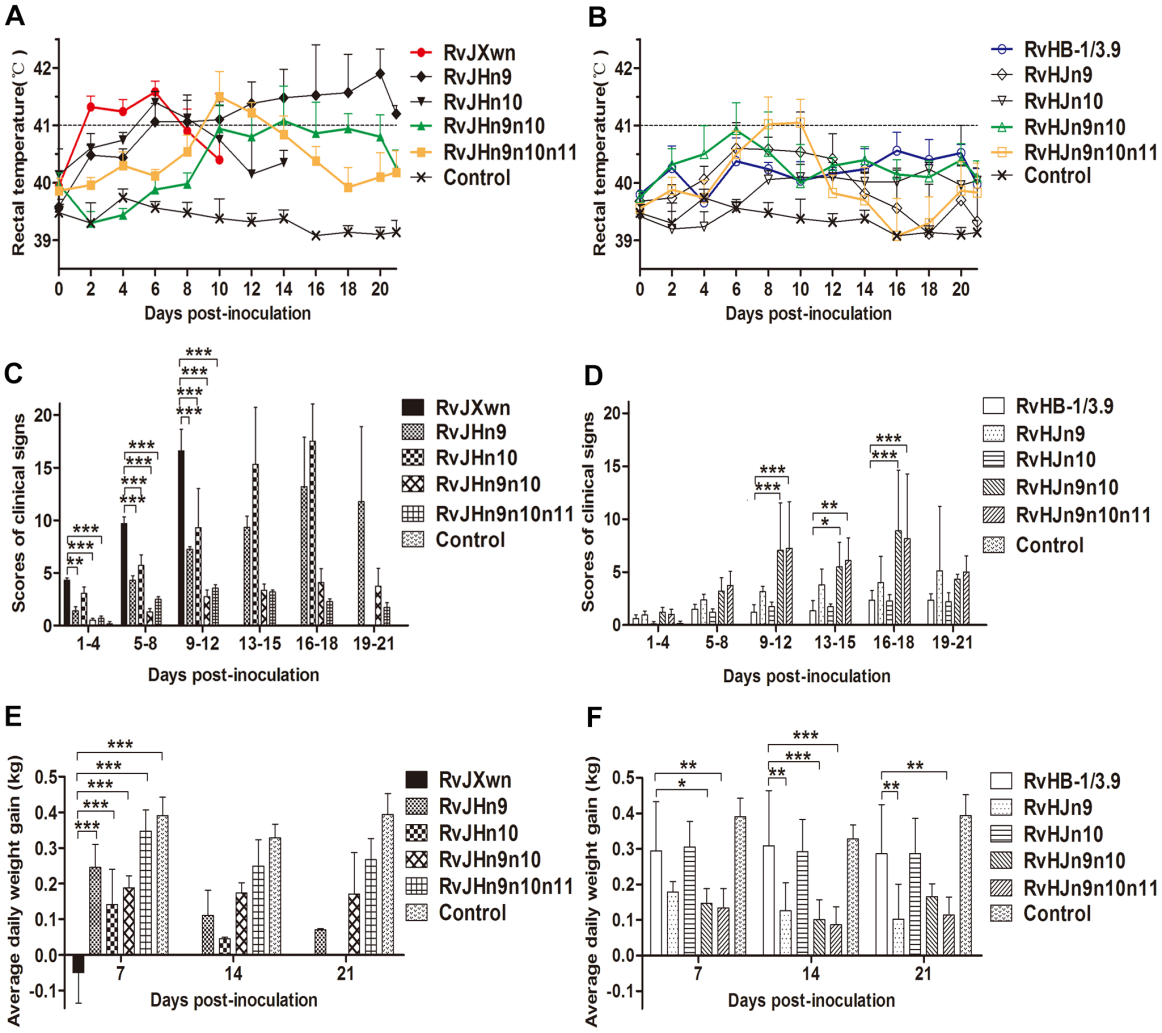
The rectal temperatures, clinical scores and average daily gains of piglets inoculated with the rescued viruses. The body temperatures, average clinical scores and average daily gains (ADG) of piglets inoculated with the rescued viruses with the RvJXwn backbone (A, C and E) or with the RvHB-1/3.9 backbone (B, D and F) are shown. The data are shown as the means ± standard deviations (error bars). The clinical scoring included the gross clinical score (GCS), respiratory clinical score (RCS) and nervous signs score (NSS). Total scores for each piglet represented the sum of the GCS, RCS and NSS. An additional five score was calculated in the total scores when the piglet died. Each piglet was scored from 0–20, and the mean values of day 1 to 4 pi, 5 to 8 pi, 9 to 12 pi, 13 to 15 pi, 16 to 18 pi and 19 to 21 pi were calculated. Asterisk indicates a significant difference between the chimeric virus and its parental backbone virus, RvJXwn or RvHB-1/3.9 (*P<0.05; **P<0.01; ***P<0.001). doi:10.1371/journal.ppat.1004216.g006

The incorrect version of Figure 7 was published. The authors have corrected the designations of rescued viruses with RvHB-1/3.9 as the backbone in the mortality table (the lower left of the figure). Please see the corrected figure here.

**Figure 7:**
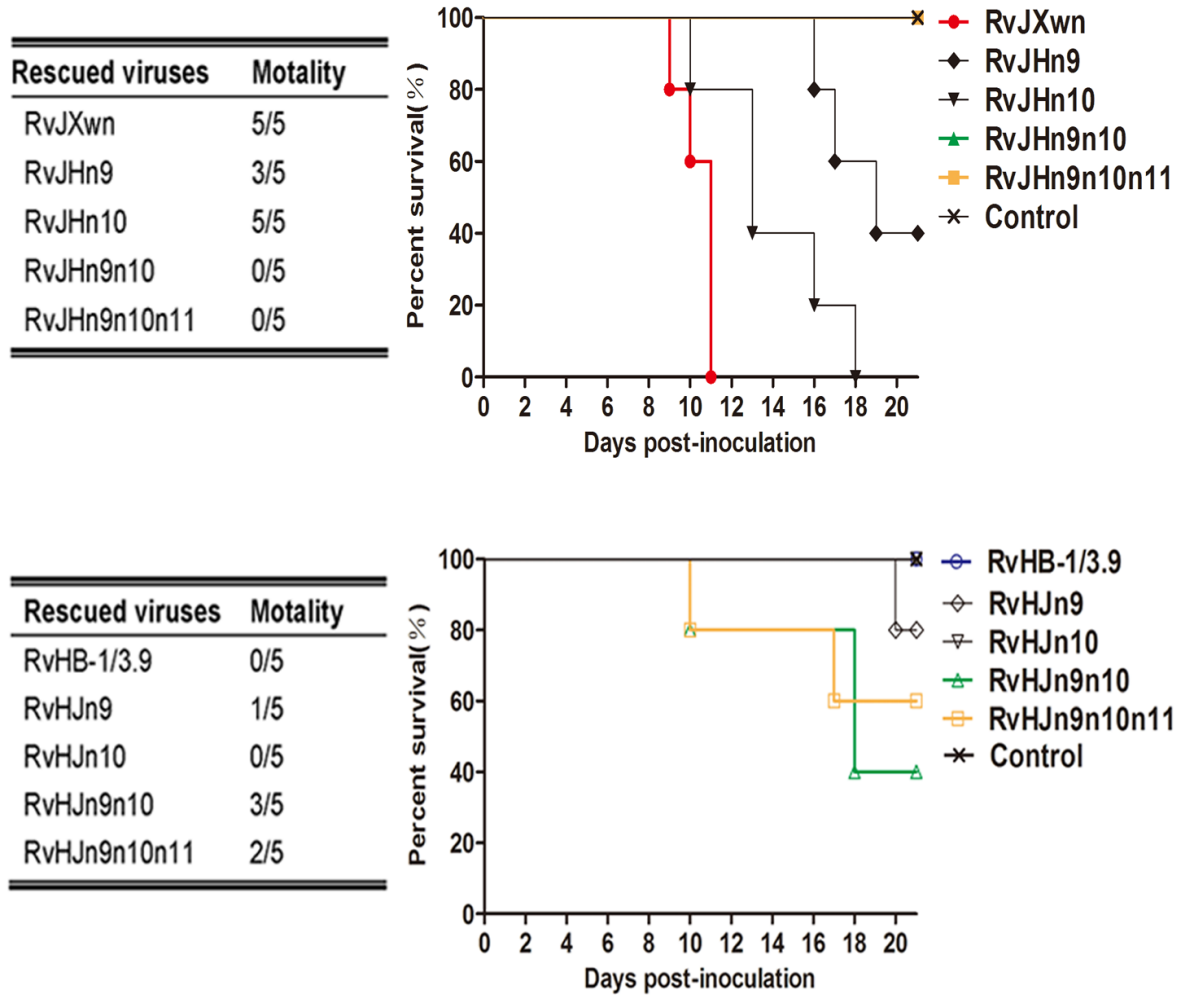
The mortality and survival curve of piglets inoculated with the rescued viruses. The mortalities and survival curves of piglets infected with rescued viruses in each group are shown (n  =  5). doi:10.1371/journal.ppat.1004216.g007
