# Supplementary figures and images for: Nsp9 and Nsp10 Contribute to the Fatal Virulence of Highly Pathogenic Porcine Reproductive and Respiratory Syndrome Virus Emerging in China
Source: PLoS Pathog. 2014 Jul 3;10(7):e1004216. doi: 10.1371/journal.ppat.1004216 (PMC4081738; doi:10.1371/journal.ppat.1004216)

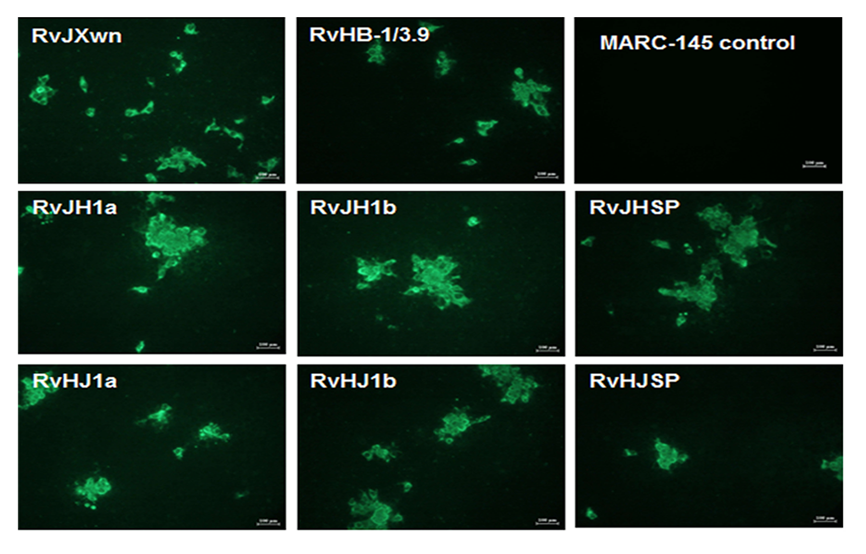

Supplement: Figure S1 — Examination of the chimeric viruses with the swapped three large coding regions. MARC-145 cells infected with a third-passage culture of the rescued viruses were fixed at 48 h postinoculation and examined by IFA using monoclonal antibodies (SDOW17) against the N protein of PRRSV. (TIF) [file ppat.1004216.s001.tif]

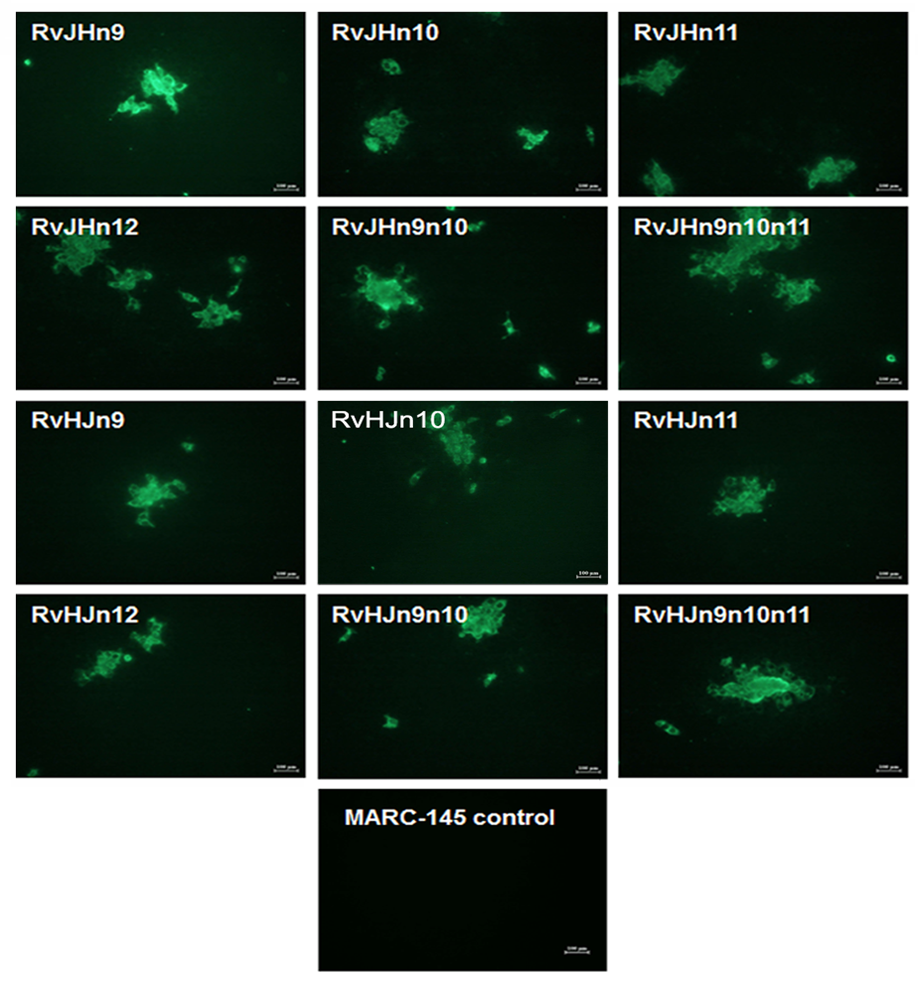

Supplement: Figure S2 — Examination of the chimeric viruses with the exchanged Nsp-coding regions within the ORF1b. MARC-145 cells infected with a third-passage culture of the rescued viruses were fixed at 48 h postinoculation and examined by IFA using monoclonal antibodies (SDOW17) against the N protein of PRRSV. (TIF) [file ppat.1004216.s002.tif]

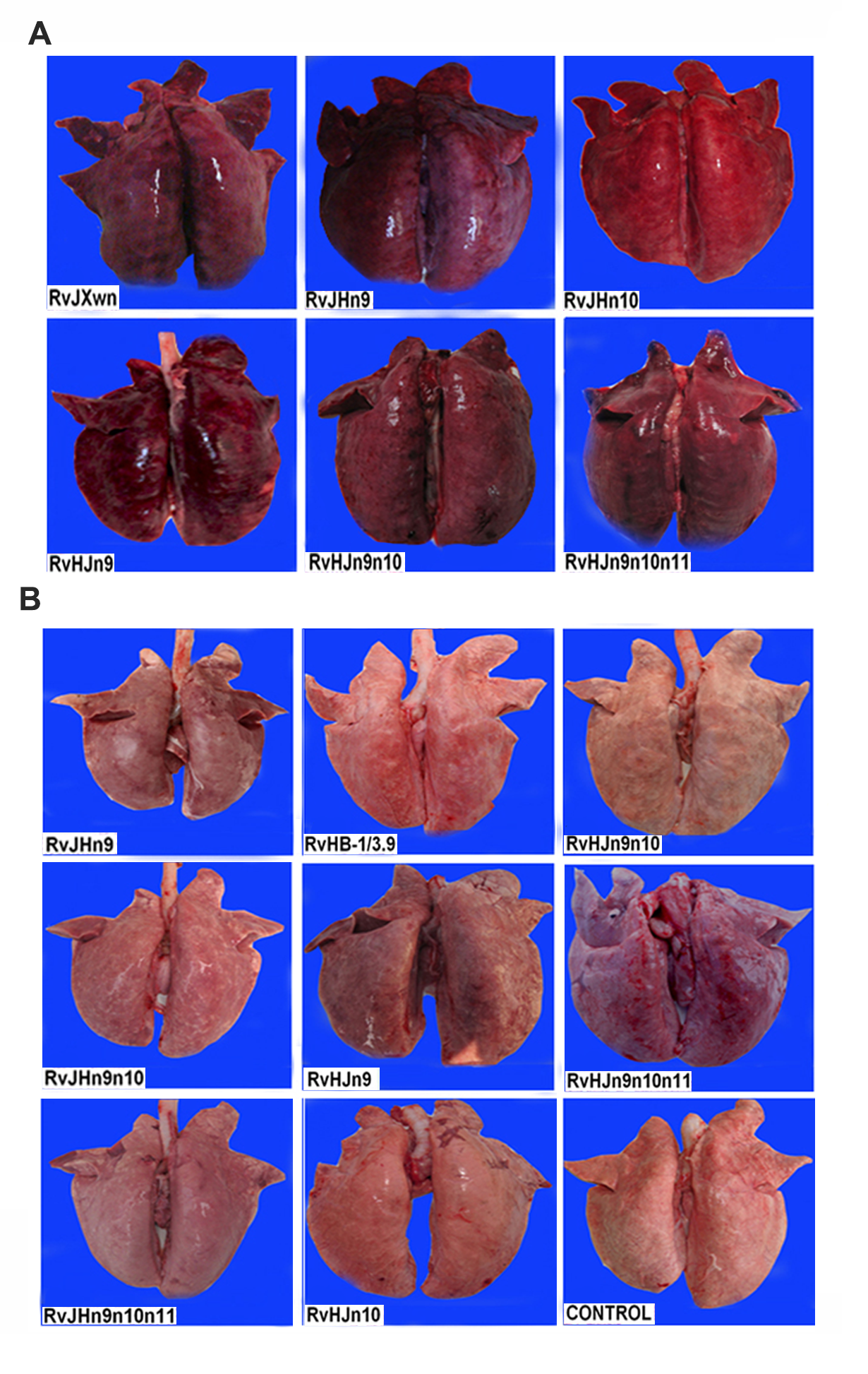

Supplement: Figure S3 — Gross lung lesions of piglets inoculated with the rescued viruses. Shown are the gross lesions of lungs from dead piglets during the experiment (A) and from euthanized piglets by the end of experiment (B) in each group. (TIF) [file ppat.1004216.s003.tif]

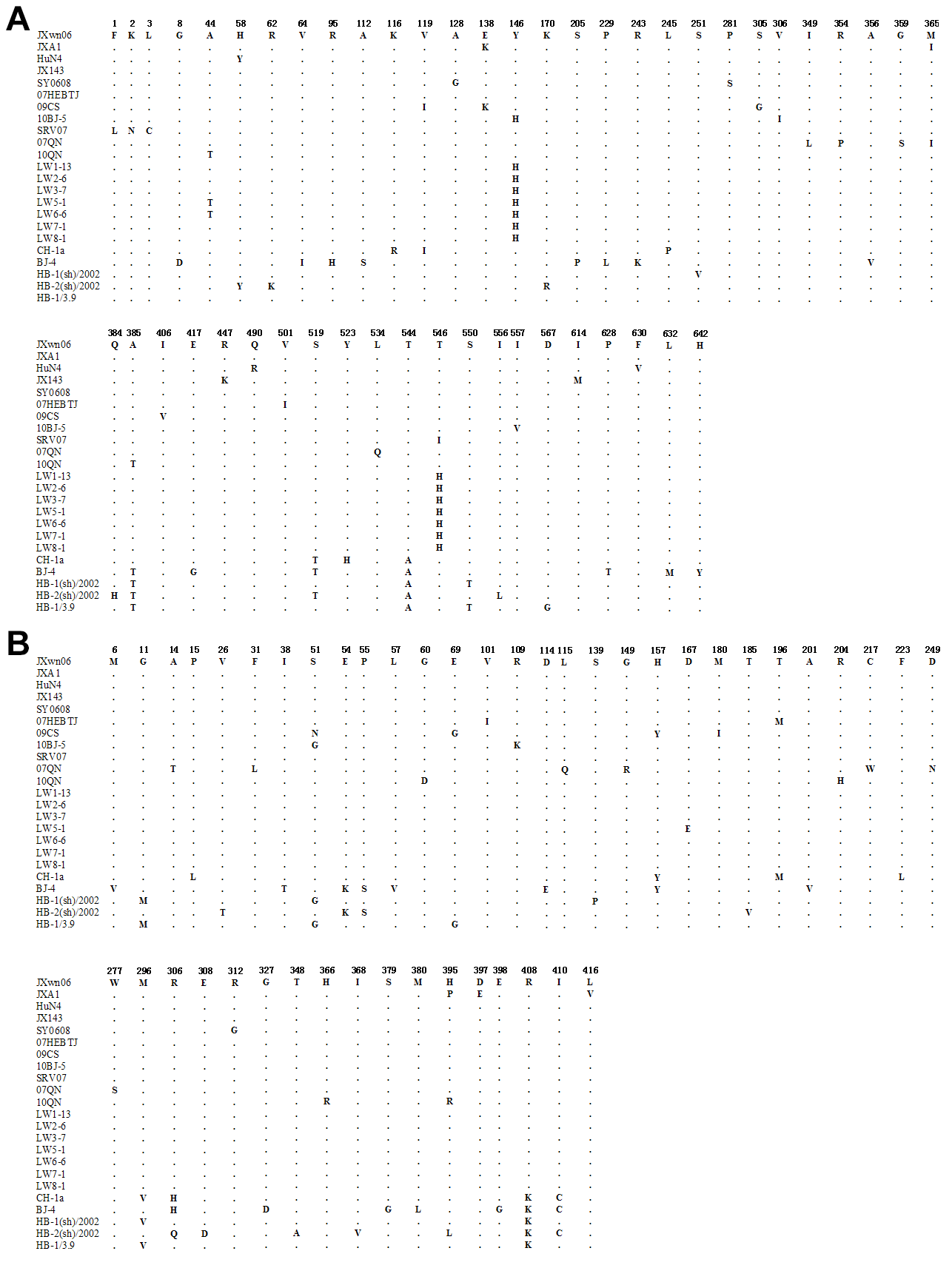

Supplement: Figure S4 — Alignment of amino acids in the Nsp9 and Nsp10 of PRRSV. (A) Nsp9. (B) Nsp10. Dots indicate conserved residues. The amino acid differences were determined based on the amino acid sequence of JXwn06 Nsp9 or Nsp10. (TIF) [file ppat.1004216.s004.tif]
