# Supplementary material for: Nsp9 and Nsp10 Contribute to the Fatal Virulence of Highly Pathogenic Porcine Reproductive and Respiratory Syndrome Virus Emerging in China
Source: PLoS Pathog. 2014 Jul 3;10(7):e1004216. doi: 10.1371/journal.ppat.1004216 (PMC4081738; doi:10.1371/journal.ppat.1004216)
Supplement: Table S1 — Primers used for construction and detection of the chimeric viruses. a F denotes a forward PCR primer; R denotes reverse transcription or a reverse PCR primer. b Numbers refer to nucleotide positions within the genome of JXwn06 (GenBank accession no: EF641008) or HB-1/3.9 (GenBank accession no: EU360130), as indicated. c Restriction sites introduced by PCR are shown in boldface and specified in parentheses at the end of the sequence. (DOC) [file ppat.1004216.s005.doc]

**Table S1 Primers used for construction and detection of the chimeric viruses**

| Primer^a^ | Genome position ^b^ | Sequence (5’-3’)^c^ | Use |
| --- | --- | --- | --- |
| RvJHSP | | | |
| JHSP-S1F | 11844-11862 | AATGGTGAGGACTGGGAGG | Fragment amplification and fusion |
| JHSP-S1R | 12071-12100 | CCAAACAAAATGGCCAAAAATATGATGATA | Fragment amplification |
| JHSP-S2F | 12037-12061 | ATGCTTTCACGGAATTTCTGGTGTC | Fragment amplification |
| JHSP-S2R | 15182-15205 | ATTCTAACACTGAGGTGCCAAAGA | Fragment amplification |
| JHSP-S3F | 15160-15180 | CCTCAGCATGATGGGCTGGCAT | Fragment amplification |
| JHSP-S3R | Poly(A)_n_ | CCCGGCCGGCC**TTAATTAA**(T)_39_ (*Pac*Ⅰ) | Fragment amplification and fusion |
| RvHJSP | | | |
| HJSP-S1F | 11931-11949 | Exactly the same as JHSP-S1F | Fragment amplification and fusion |
| HJSP-S1R | 12158-12187 | Exactly the same as JHSP-S1R | Fragment amplification |
| HJSP-S2F | 12124-12148 | Exactly the same as JHSP-S2F | Fragment amplification |
| HJSP-S2R | 15269-15292 | Exactly the same as JHSP-S2R | Fragment amplification |
| HJSP-S3F | 15247-15268 | Exactly the same as JHSP-S3F | Fragment amplification |
| HJSP-S3R | Poly(A)_n_ | ATTTAAAT**CGGACCG**(T)_39_ (*Rsr*Ⅱ) | Fragment amplification and fusion |
| RvJHn9 | | | |
| HBn9-F | 7620-7649 | GACTGCCAAAGAACTGGAGAAACTGAAAAG | Fragment amplification and fusion |
| HBn9-R | 9616-9641 | GTACCCGCACATTCTGGACTTCTTCC | Fragment amplification |
| JXrC1-F | 9480-9509 | CCACCGTTCTTCTTGTCCATGTGGGAAAAA | Fragment amplification |
| JXC-R | 12385-12409 | CCAAACCACTAATGCGAGACAATGT | Fragment amplification and fusion |
| RvHJn9 | | | |
| JXn9-F | 7530-7559 | Exactly the same as HBn9-F | Fragment amplification and fusion |
| JXn9-R | 9533-9555 | AGTACCCGCACATTCTGGACTTC | Fragment amplification |
| HBrD1-F | 9577-9603 | TCTTGTCCATGTGGGAAAAACTCAGGT | Fragment amplification |
| HBD-R | 12037–12061 | AGGCCTAAAGTTGGTTCAATGACAG | Fragment amplification and fusion |
| RvJHn10 | | | |
| JXn9-F | 7530-7559 | Exactly the same as HBn9-F | Fragment amplification and fusion |
| JXn9-R | 9533-9555 | AGTACCCGCACATTCTGGACTTC | Fragment amplification |
| HBn10-F | 9577-9603 | Exactly the same as HBrD1-F | Fragment amplification |
| HBn10-R | 10941-10970 | ATGCGCGACCTTGGGGAGCGGGGAGCTCGA | Fragment amplification |
| JXrC2-F | 10853-10883 | TCGAGCTCCCCGCTCCCCAAGGTCGCGCAT | Fragment amplification |
| JXC-R | 12385-12409 | CCAAACCACTAATGCGAGACAATGT | Fragment amplification and fusion |
| RvHJn10 | | | |
| HBn9-F | 7620-7649 | GACTGCCAAAGAACTGGAGAAACTGAAAAG | Fragment amplification and fusion |
| HBn9-R | 9616-9641 | GTACCCGCACATTCTGGACTTCTTCC | Fragment amplification |
| JXn10-F | 9480-9509 | Exactly the same as JXrC1-F | Fragment amplification |
| JXn10-R | 10854-10884 | Exactly the same as HBn10-R | Fragment amplification |
| HBrD2-F | 10941-10970 | Exactly the same as JXrC2-F | Fragment amplification |
| HBD-R | 12037–12061 | AGGCCTAAAGTTGGTTCAATGACAG | Fragment amplification and fusion |
| RvJHn11 | | | |
| JXn9n10-F | 7530-7559 | Exactly the same as HBn9-F | Fragment amplification and fusion |
| JXn9n10-R | 10854-10884 | Exactly the same as HBn10-R | Fragment amplification |
| HBn11-F | 10941-10970 | Exactly the same as JXrC2-F | Fragment amplification |
| HBn11-R | 11596-11627 | ATACCAGGTAAAATGGCGGCCTTCAAGTTGAA | Fragment amplification |
| JXrC3-F | 11509-11540 | TTCAACTTGAAGGCCGCCATTTTACCTGGTAT | Fragment amplification |
| JXC-R | 12385-12409 | CCAAACCACTAATGCGAGACAATGT | Fragment amplification and fusion |
| RvHJn11 | | | |
| HBn9n10-F | 7620-7649 | Exactly the same as HBn9-F | Fragment amplification and fusion |
| HBn9n10-R | 10941-10970 | Exactly the same as HBn10-R | Fragment amplification |
| JXn11-F | 10853-10883 | Exactly the same as JXrC2-F | Fragment amplification |
| JXn11-R | 11509-11540 | Exactly the same as HBn11-R | Fragment amplification |
| HBrD3-F | 11596-11627 | Exactly the same as JXrC3-F | Fragment amplification |
| HBD-R | 12037–12061 | AGGCCTAAAGTTGGTTCAATGACAG | Fragment amplification and fusion |
| RvJHn12 | | | |
| JXn9n10n11-F | 7530-7559 | Exactly the same as HBn9-F | Fragment amplification and fusion |
| JXn9n10n11-R | 11509-11540 | Exactly the same as HBn11-R | Fragment amplification |
| HBn12-1F | 11596-11627 | Exactly the same as JXrC3-F | Fragment amplification |
| JXC-R | 12385-12409 | CCAAACCACTAATGCGAGACAATGT | Fragment amplification and fusion |
| HBn12-2F | 11632-11664 | TTGCAAGTTACGCCTCATACATCCGAGTTCCTG | Fragment amplification and fusion |
| HBn12-2R | 12061-12090 | TTTGCATAGACCCCATTTCA TTTCAATTCA | Fragment amplification |
| JXrD-F | 11974-12003 | TGAATTGAAATGAAATGGGGTCTATGCAAA | Fragment amplification |
| JXD-R | Poly(A)_n_ | Exactly the same as JHSP-S3R | Fragment amplification and fusion |
| RvHJn12 | | | |
| HBn9n10n11-F | 7620-7649 | Exactly the same as HBn9-F | Fragment amplification and fusion |
| HBn9n10n11-R | 11596-11627 | Exactly the same as HBn11-R | Fragment amplification |
| JXn12-1F | 11509-11540 | TTCAACTTGAAGGCCGCCATTTTACCTGGTAT | Fragment amplification |
| HBD-R | 12037–12061 | AGGCCTAAAGTTGGTTCAATGACAG | Fragment amplification and fusion |
| JXn12-2F | 11844-11863 | AATGGTGAGGACTGGGAGGA | Fragment amplification and fusion |
| JXn12-2R | 12078-12101 | GCCAAACAAAATGGCCAAAA ATAT | Fragment amplification |
| HBrE-1F | 12127-12147 | CTTTCACGGAATTTCTGGTGT | Fragment amplification |
| HBrE-1R | 12903-12923 | TATCCTGCACCAAAGAGACCT | Fragment amplification |
| HBrE-2F | 12781-12802 | CTACGTTCTGTTTTTGGTTTCC | Fragment amplification |
| HBE-R | Poly(A)_n_ | Exactly the same as HJSP-S3R | Fragment amplification and fusion |
| RvJHn9n10 | | | |
| HBn9n10-F | 7620-7649 | Exactly the same as HBn9-F | Fragment amplification and fusion |
| HBn9n10-R | 10941-10970 | Exactly the same as HBn10-R | Fragment amplification |
| JXrC2-F | 10853-10883 | TCGAGCTCCCCGCTCCCCAAGGTCGCGCAT | Fragment amplification |
| JXC-R | 12385-12409 | CCAAACCACTAATGCGAGACAATGT | Fragment amplification and fusion |
| RvHJn9n10 | | | |
| JXn9n10-F | 7530-7559 | Exactly the same as HBn9-F | Fragment amplification and fusion |
| JXn9n10-R | 10854-10884 | Exactly the same as HBn10-R | Fragment amplification |
| HBrD2-F | 10941-10970 | Exactly the same as JXrC2-F | Fragment amplification |
| HBD-R | 12037–12061 | AGGCCTAAAGTTGGTTCAATGACAG | Fragment amplification and fusion |
| RvJHn9n10n11 | | | |
| HBn9n10n11-F | 7620-7649 | Exactly the same as HBn9-F | Fragment amplification and fusion |
| HBn9n10n11-R | 11596-11627 | Exactly the same as HBn11-R | Fragment amplification |
| JXrC3-F | 11509-11540 | TTCAACTTGAAGGCCGCCATTTTACCTGGTAT | Fragment amplification |
| JXC-R | 12385-12409 | CCAAACCACTAATGCGAGACAATGT | Fragment amplification and fusion |
| RvHJn9n10n11 | | | |
| JXn9n10n11-F | 7530-7559 | Exactly the same as HBn9-F | Fragment amplification and fusion |
| JXn9n10n11-R | 11509-11540 | Exactly the same as HBn11-R | Fragment amplification |
| HBrD3-F | 11596-11627 | Exactly the same as JXrC3-F | Fragment amplification |
| HBD-R | 12037–12061 | AGGCCTAAAGTTGGTTCAATGACAG | Fragment amplification and fusion |
| N312-F | 14860-14881 | AGCTGTGCCAAATGCTGGGTAA | ORF7 detection |
| N312-R | 15150-15171 | ATCATGCTGAGGGTGATGCTGT | ORF7 detection |
| Detect-F | 2497–2517 | CTTAAAGACCAGATGGAGGAGG | Nsp2 detection |
| Detect-R | 3156–3178 | CGATGATGGCTTGAGCTGAGTAT | Nsp2 detection |

^a^ F denotes a forward PCR primer; R denotes reverse transcription or a reverse PCR primer.

^b^ Numbers refer to nucleotide positions within the genome of JXwn06 (GenBank accession no: EF641008) or HB-1/3.9 (GenBank accession no: EU360130), as indicated.

^c^ Restriction sites introduced by PCR are shown in boldface and specified in parentheses at the end of the sequence.

|  |  |  |  |
| --- | --- | --- | --- |
|  |  |  |  |
|  |  |  |
|  |  |  |  |
|  |  |  |  |
|  |  |  |  |
|  |  |  |  |
|  |  |  |  |
|  |  |  |  |
|  |  |  |  |
|  |  |  |  |
|  |  |  |
|  |  |  |  |
|  |  |  |  |
|  |  |  |  |
|  |  |  |  |
|  |  |  |  |
|  |  |  |  |
|  |  |  |  |
|  |  |  |  |
|  |  |  |
|  |  |  |  |
|  |  |  |  |
|  |  |  |  |

Usual condition
