# Supplementary material for: Nsp9 and Nsp10 Contribute to the Fatal Virulence of Highly Pathogenic Porcine Reproductive and Respiratory Syndrome Virus Emerging in China
Source: PLoS Pathog. 2014 Jul 3;10(7):e1004216. doi: 10.1371/journal.ppat.1004216 (PMC4081738; doi:10.1371/journal.ppat.1004216)
Supplement: Table S2 — Clinical sign scoring system used for analyzing the pathogenicity of rescued viruses. Usual condition: total score = GCS+RCS+NSS. If piglet died: total score = GCS+RCS+NSS+5. 0≤total score≤20. (DOCX) [file ppat.1004216.s006.docx]

**Table S2 Clinical signs scoring system used for analyzing pathogenicity of the rescued viruses**

|  | Clinical signs | Criteria [for](app:ds:for) [evaluation](app:ds:evaluation) | Score |
| --- | --- | --- | --- |
| Gross clinical score (GCS) | ①Body temperature | T ≤39.9℃ | 0 |
|  |  | 40.0℃≤T≤40.9℃ | 1 |
|  |  | 41.0℃≤T | 2 |
|  | ②Appetite | Normal | 0 |
|  |  | Inappetence | 1 |
|  | ③Awareness | Normal | 0 |
|  |  | Lethargic | 1 |
|  | ④Skin | Normal | 0 |
|  |  | Rubefaction | 1 |
| Respiratory clinical score (RCS) | ①Respiratory condition | Normal | 0 |
|  |  | Tachypnea (nervous) | 1 |
|  |  | Tachypnea (calm) | 2 |
|  |  | Tachypnea and dyspnea | 3 |
|  |  | Tachypnea, dyspnea and Irregular respiration | 4 |
|  | ②Cough | Normal | 0 |
|  |  | Cough | 1 |
|  | ③Rhinorrhoea | Normal | 0 |
|  |  | Rhinorrhoea | 1 |
| Nervous signs score (NSS) | ①Nervous signs | Normal | 0 |
|  |  | Shiver | 1 |
|  |  | Ataxia | 2 |
|  |  | Incoordination | 3 |
|  |  | Paralysis | 4 |

Usual condition: total score = GCS+RCS+NSS

If piglet died: total score = GCS+RCS+NSS+5

0 ≤ total score ≤ 20
